# Micronutrients, Vitamin D, and Inflammatory Biomarkers in COVID-19: A Systematic Review and Meta-analysis of Causal Inference Studies

**DOI:** 10.1093/nutrit/nuae152

**Published:** 2024-10-24

**Authors:** Ángela Alcalá-Santiago, Miguel Rodriguez-Barranco, María-José Sánchez, Ángel Gil, Belén García-Villanova, Esther Molina-Montes

**Affiliations:** Department of Nutrition and Food Science, Faculty of Pharmacy, University of Granada, 18071 Granada, Spain; Instituto de Investigación Biosanitaria ibs.Granada, 18012 Granada, Spain; Institute of Nutrition and Food Technology (INYTA) “José Mataix”, Biomedical Research Centre, University of Granada, 18071 Granada, Spain; Instituto de Investigación Biosanitaria ibs.Granada, 18012 Granada, Spain; CIBER of Epidemiology and Public Health (CIBERESP), 28029 Madrid, Spain; Andalusian School of Public Health, 18012 Granada, Spain; Instituto de Investigación Biosanitaria ibs.Granada, 18012 Granada, Spain; CIBER of Epidemiology and Public Health (CIBERESP), 28029 Madrid, Spain; Andalusian School of Public Health, 18012 Granada, Spain; Instituto de Investigación Biosanitaria ibs.Granada, 18012 Granada, Spain; Institute of Nutrition and Food Technology (INYTA) “José Mataix”, Biomedical Research Centre, University of Granada, 18071 Granada, Spain; Department of Biochemistry and Molecular Biology II, Faculty of Pharmacy, University of Granada, 18071 Granada, Spain; CIBER de Obesidad y Nutrición (CIBEROBN), 28029 Madrid, Spain; Department of Nutrition and Food Science, Faculty of Pharmacy, University of Granada, 18071 Granada, Spain; Department of Nutrition and Food Science, Faculty of Pharmacy, University of Granada, 18071 Granada, Spain; Instituto de Investigación Biosanitaria ibs.Granada, 18012 Granada, Spain; Institute of Nutrition and Food Technology (INYTA) “José Mataix”, Biomedical Research Centre, University of Granada, 18071 Granada, Spain; CIBER of Epidemiology and Public Health (CIBERESP), 28029 Madrid, Spain

**Keywords:** Mendelian randomization, causal inference, vitamin D, micronutrients, inflammatory markers

## Abstract

**Context:**

Experimental and observational studies suggest that circulating micronutrients, including vitamin D (VD), may increase COVID-19 risk and its associated outcomes. Mendelian randomization (MR) studies provide valuable insight into the causal relationship between an exposure and disease outcomes.

**Objectives:**

The aim was to conduct a systematic review and meta-analysis of causal inference studies that apply MR approaches to assess the role of these micronutrients, particularly VD, in COVID-19 risk, infection severity, and related inflammatory markers.

**Data Sources:**

Searches (up to July 2023) were conducted in 4 databases.

**Data Extraction and Analysis:**

The quality of the studies was evaluated based on the MR-STROBE guidelines. Random-effects meta-analyses were conducted where possible.

**Results:**

There were 28 studies (2 overlapped) including 12 on micronutrients (8 on VD) and COVID-19, 4 on micronutrients (all on VD) and inflammation, and 12 on inflammatory markers and COVID-19. Some of these studies reported significant causal associations between VD or other micronutrients (vitamin C, vitamin B_6_, iron, zinc, copper, selenium, and magnesium) and COVID-19 outcomes. Associations in terms of causality were also nonsignificant with regard to inflammation-related markers, except for VD levels below 25 nmol/L and C-reactive protein (CRP). Some studies reported causal associations between cytokines, angiotensin-converting enzyme 2 (ACE2), and other inflammatory markers and COVID-19. Pooled MR estimates showed that VD was not significantly associated with COVID-19 outcomes, whereas ACE2 increased COVID-19 risk (MR odds ratio = 1.10; 95% CI: 1.01–1.19) but did not affect hospitalization or severity of the disease. The methodological quality of the studies was high in 13 studies, despite the majority (n = 24) utilizing 2-sample MR and evaluated pleiotropy.

**Conclusion:**

MR studies exhibited diversity in their approaches but do not support a causal link between VD/micronutrients and COVID-19 outcomes. Whether inflammation mediates the VD–COVID-19 relationship remains uncertain, and highlights the need to address this aspect in future MR studies exploring micronutrient associations with COVID-19 outcomes.

**Systematic Review Registration:**

PROSPERO registration no. CRD42022328224.

## Graphical Abstract

**Figure:**
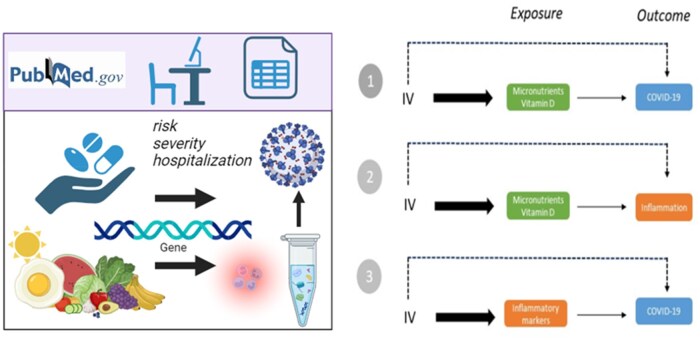

## INTRODUCTION

COVID-19, caused by severe acute respiratory syndrome coronavirus 2 (SARS-CoV-2), was declared a global pandemic by the World Health Organization (WHO) in 2020.^1^ This virus poses a higher risk to elderly individuals, who are more prone to severe outcomes and mortality. Their risk is further increased in the presence of multiple comorbidities, such as diabetes, cardiovascular disease, respiratory diseases, cancer, and obesity.^2^^,^^3^

Several research studies have demonstrated that specific micronutrients, including vitamins (A, B_9_, B_12_, C, D, and E) and minerals (zinc, selenium, iron, and copper), are involved in the proper functioning and regulation of the immune system.^4^ These micronutrients (which are included in the so-called group of immunonutrients) also act as epigenetic modifiers that block the immune response in inflammatory processes. Interestingly, SARS-CoV-2 virus infection has been found to deplete stores of various micronutrients, with a particular emphasis on vitamin D (VD).^5^

Adequate VD levels (25-hydroxyvitamin D [25(OH)D]) are determined by the body’s synthesis through sunlight exposure and, although to a lesser extent, approximately 10%–20% of the total VD^6^ by intake of VD from dietary sources and supplements. There are 2 main forms of VD found in the diet: cholecalciferol (vitamin D_3_), derived from animal sources, and ergocalciferol (vitamin D_2_), found in certain fungi and plants. Fatty fish, fortified dairy products, and certain mushrooms are the most important sources of VD.^7^ Beyond its well-known role in maintaining bone health, VD has several immunomodulatory effects. More precisely, this nutrient participates in both innate immunity, promoting the production of antimicrobial peptides such as cathelicidins, defensins, and interleukin (IL)-37, as well as adaptive immunity by regulating key proinflammatory cytokines (eg, IL-6, tumor necrosis factor [TNF]-alpha, and interferon-gamma) and controlling the immune response mediated by T-helper 1 (Th1) lymphocytes.^8^ Low levels of 25(OH)D, below which deficiency has been found to exist, seem to be genetically driven and are known to cause inflammation and several comorbid conditions, including metabolic diseases and cancer.^9^

Some studies have shown that there is an association between 25(OH)D levels and different clinical outcomes of COVID-19, particularly in relation to its severity and mortality.^10–12^ There are several mechanisms that appear to underlie this effect. For instance, it has been demonstrated that, upon entering respiratory epithelial cells, the SARS-CoV-2 virus triggers an immune response leading to the production of inflammatory cytokines, followed by the infiltration of macrophages and neutrophils into lung tissue, which, in turn, results in a cytokine storm that causes widespread inflammation and aggravates the disease.^13^ A deficiency in VD and other micronutrients is associated with systemic inflammation, as evidenced by elevated levels of C-reactive protein (CRP), which can further amplify the inflammatory response caused by this virus.^14^

On the other hand, the SARS-CoV-2 virus binds to host receptors through S glycoproteins, facilitating viral penetration. The primary receptor for this virus is the membrane angiotensin-converting enzyme 2 (ACE2).^15^ Aside from enabling virus entry, this receptor also leads to downregulation of anti-inflammatory ACE2 expression, resulting in an excess of proinflammatory angiotensin II through the ACE enzyme. Chronic overstimulation of the renin-angiotensin system (RAS) induces undesirable effects, such as inflammation, oxidative stress, and myocardial hypertrophy. The ACE system has been attributed to various pathophysiological processes associated with the severity and progression of COVID-19. Vitamin D regulates the expression of renin and interacts with the RAS/ACE/ACE-2 signaling axis, counteracting the negative effects of ACE.^16^ Other micronutrients also seem to interact with the ACE system.^17^

In light of the multiple roles that micronutrients play in immune function and their potential influence on the severity and outcome of COVID-19, understanding the impact of these micronutrients and their supplementation has become a topic of significant scientific interest.^10–12^ Proper nutrition and ensuring sufficient intake of these micronutrients may have implications for enhancing immune responses and mitigating the effects of the virus in vulnerable populations.

Mendelian randomization (MR) studies can provide evidence on the causal link between micronutrients and COVID-19 and related markers, using genetic variants associated with an exposure of interest as an instrumental variable (IV). Since there is a random allocation of genetic variants at conception, causal estimates can be derived as in randomized controlled trials. Thus, the MR study design offers numerous advantages over traditional observational studies. The genetic variants are identified through genome-wide association studies (GWASs). To comply with MR assumptions, the chosen genetic variants must be associated with the exposure but not linked to any confounding factor in the exposure–outcome relationship, nor should they be associated with the outcome through any pathway other than the exposure of interest. These 3 assumptions constitute the definition of an IV in MR analyses.^18^ Mendelian randomization analysis can be conducted using existing datasets on gene–phenotype associations, making it a highly efficient approach.^19^ Consequently, in recent years, the literature has seen a substantial increase in the number of MR studies, among which some have focused on COVID-19,^20^^,^^21^ on VD,^14^^,^^22^ or on micronutrients.^23^^,^^24^ To the best of available knowledge, no study has attempted to summarize the evidence provided by MR studies on the potential of VD and other micronutrients for preventing and treating COVID-19 disease.

In this context, this study aimed to conduct a systematic review of MR studies to evaluate the causal role of these micronutrients on COVID-19 disease, on the inflammatory state, and on their effects on ACE2.

## METHODS

### Study Design

A systematic literature review was performed in accordance with the Preferred Reporting Items for Systematic Reviews and Meta-Analyses (PRISMA) statement.^25^ The protocol of the systematic review was registered at the International Prospective Register of Systematic Reviews (PROSPERO: CRD42022328224).

### Sources of Information and Study Selection

Mendelian randomization studies that evaluated associations of genetically predicted exposures with COVID-19 and inflammation-related outcomes were considered eligible in this review. The exposures considered were micronutrients, VD, and inflammatory-related markers, including ACE2.

Two reviewers (A.A.-S. and E.M.-M.) independently searched the Web of Science (WOS), Cochrane, Scopus, and MEDLINE databases for published studies from inception to July 17, 2023. Different search strategies were used using key words such as micronutrients, VD, COVID-19, inflammation biomarkers, ACE2, and MR. Reference lists of retrieved studies were also hand-searched to identify additional studies. The search strategies that were used are defined in the **Supplementary Material**.

The titles and abstracts of all retrieved manuscripts were reviewed to identify the studies that met the selection criteria outlined in a predetermined PICO (Population, Intervention, Comparison, Outcomes) framework, as shown in **Table 1**.

**Table 1.** PICOS Criteria for Inclusion of Studies

Parameter	Criterion
P (Population)	Men or women, adults
I (Intervention)	Levels of micronutrients, vitamin D and inflammatory-related markers, alone or in combination
C (Comparison)	Normal levels versus deficient levels of micronutrients, vitamin D and inflammatory-related markers
O (Outcomes)	Studies accounting for the severity and progression of COVID-19 or inflammatory-related markers

In addition, other inclusion criteria considered were as follows:

- Studies focusing on exposure–outcome associations, conducted among adults
- Studies assessing causal associations through MR approaches
- Studies accounting for the severity and progression of COVID-19 or inflammatory-related markers

**Figure 1.**
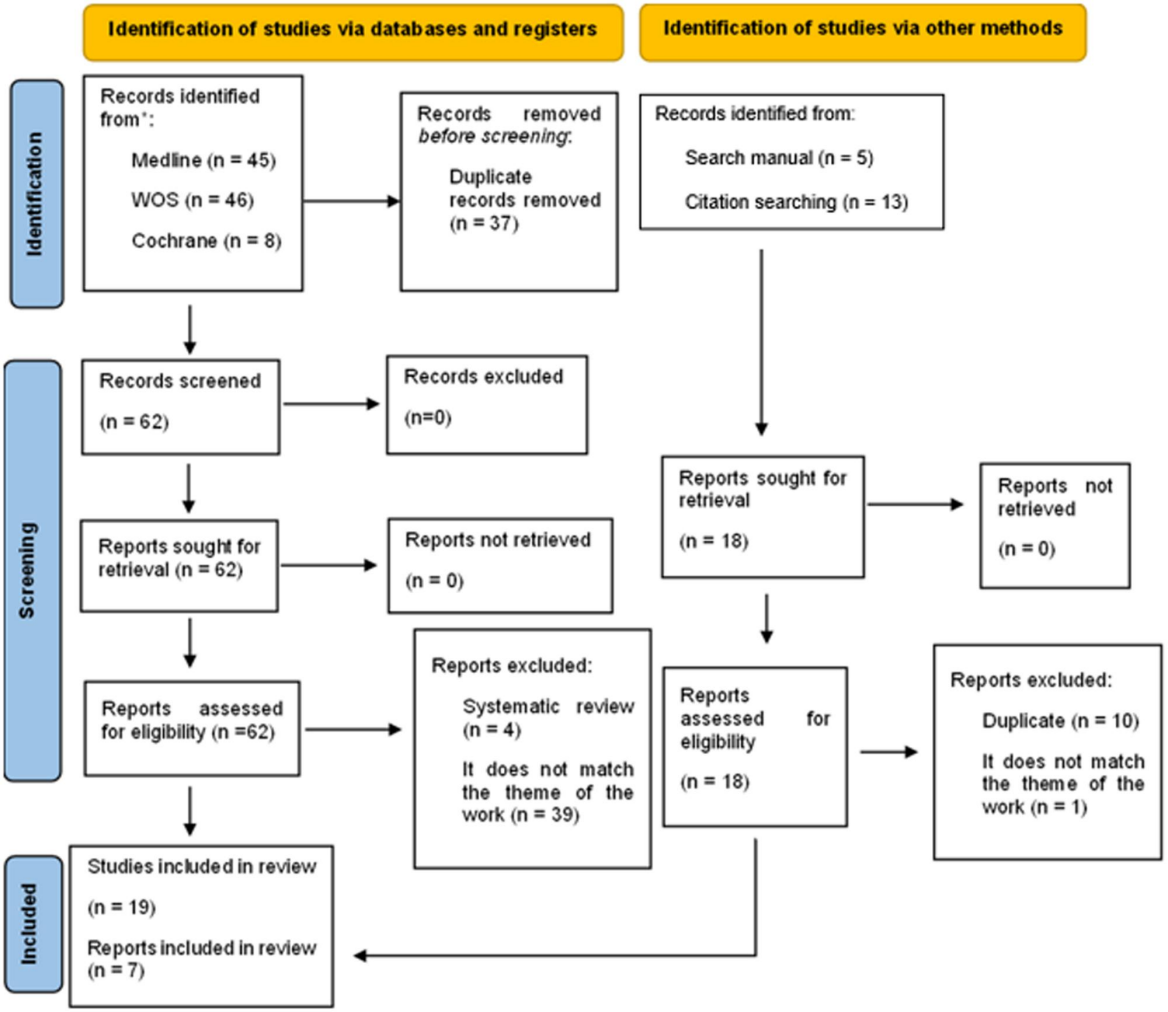
PRISMA (Preferred Reporting Items for Systematic Reviews and Meta-Analyses) 2020 Flow Diagram of the Study Search and Selection Process.^24^ Abbreviation: WOS, Web of Science

Subsequently, each manuscript was reviewed by both reviewers in full text to confirm eligibility for inclusion, and inconsistencies were resolved by consensus or involving a third researcher (B.G.-V. or M.R.-B.). Studies that were duplicated in the different databases, those lacking original data (eg, reviews, comments, corrections, and summaries), those that were not MR studies, or those that did not include COVID-19 phenotypes or inflammation markers (ie, not meeting the inclusion criteria) were excluded. The article language was not a selection criterion, as all papers had abstracts written in English. The results of the selection of articles for this review are presented in a PRISMA flowchart (**Figure 1**).^26^

### Data Extraction

One of the main authors (A.A.-S.) extracted key information from each manuscript. These included the name of the first author, the year of publication, the ancestry of the genetic variants, characteristics of the study population (number of participants considered to build the IV associated with the exposure and number of participants considered for the outcome assessment) and data sources, the number of genetic variants, the exposure, the results, and the type of MR study and methods (1-sample or 2-sample, Egger, etc). The effect estimates, the 95% CIs, and the *P* values from the main analysis were extracted, as reported in the main text. The extracted data were verified by another reviewer (E.M.-M.).

### Methodological Quality Assessment (Risk of Bias)

The risk of bias was evaluated using the guidelines proposed by Burgess et al,^27^ and those defined by STROBE (Strengthening the Reporting of Observational Studies in Epidemiology) for MR studies.^28^ Given that these tools are not structured as checklists, the guidelines were adapted into a checklist format, so as to be able to assess the methodological quality of each study. The aforementioned MR guidelines comprised 20 items and 30 subitems, all of which should be addressed when reporting an MR study. Those items that focused on the methods and reporting of results and discussion were considered in the checklist (*n* = 28). Yes (1 point), no (0 points), unclear or NA (0 points) were assigned, and considered the study quality to be high when a score (range varying between 0 and 28 points) higher than 75% was reached (ie, >21 of 28 points) and low for scores below 75% (ie, <21 of 28 points).

Data extraction and quality assessment were independently performed by 2 researchers (A.A.-S. and E.M.-M.), and inconsistencies were resolved by consensus or involving a third researcher (B.G.-V. and M.R.-B.).

### Data Synthesis

**Figure 2.**
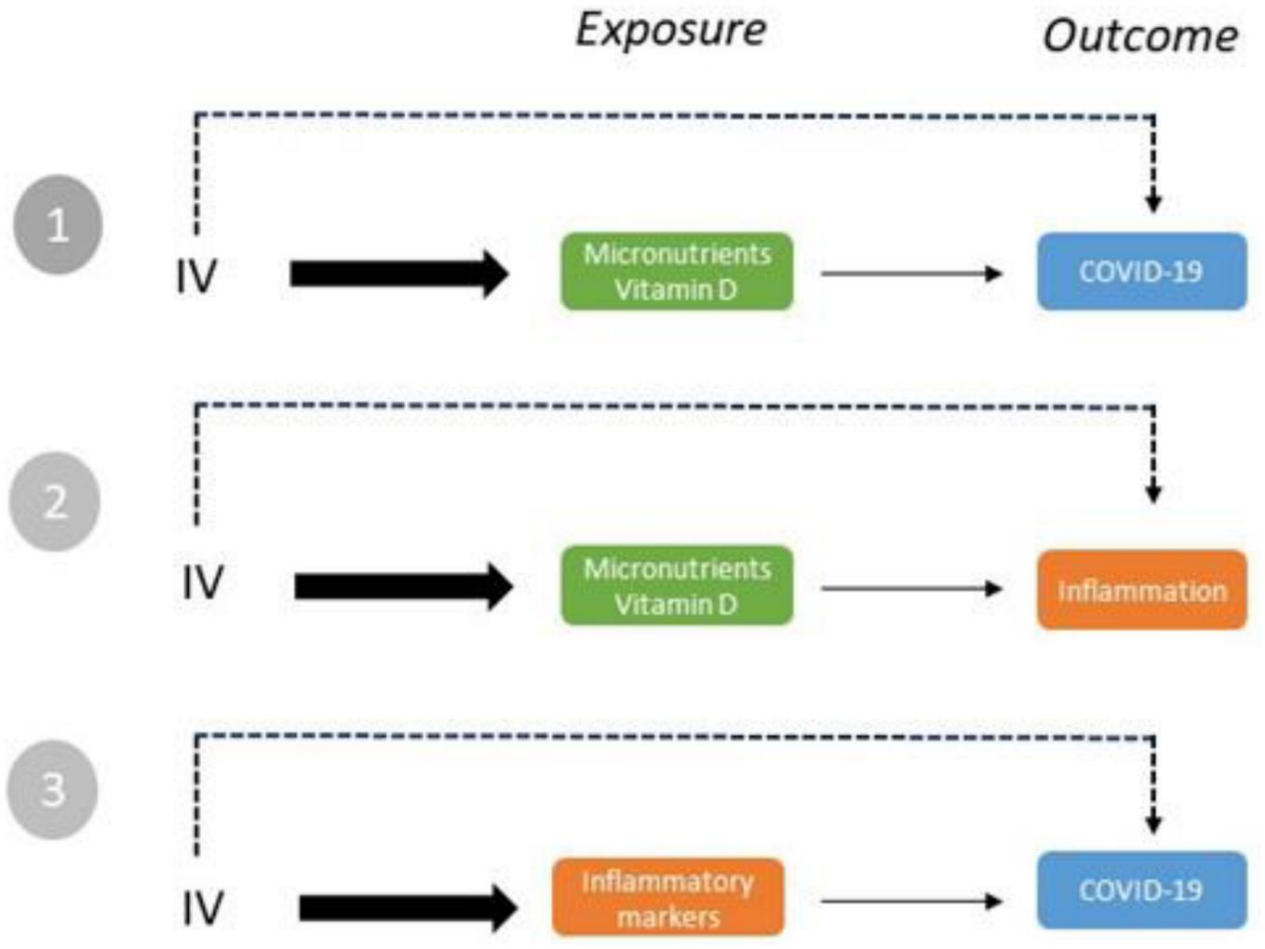
Exposure–Outcome Associations. Mendelian randomization (MR) studies considering instrumental variables (IVs) defined by micronutrients and vitamin D (VD; 1 and 2) or inflammatory markers (3) and studies that further assessed the association between IVs and COVID-19 (1 and 3) or inflammatory markers (2) were considered in this review. The main MR assumptions are indicated. The IV is associated with the exposure, and the association of the IV with the outcome occurs through the exposure

The evidence was summarized qualitatively by the exposure–outcome associations that were defined (as detailed in **Figure 2**): VD, micronutrients, inflammation markers, and COVID-19. For the latter, the results were synthesized by disease severity and progression. Levels of VD status were also considered for data synthesis. Due to the overlap of participants among MR studies (data sources were common to several studies), a quantitative meta-analysis was undertaken where possible. Random-effects models were applied to pool summary statistics (odds ratios [ORs] and SEs) of the studies. Potential heterogeneity was explored by the *I^2^* statistic, and publication bias was explored by funnel plots. The R software (version 4.2.2; R Foundation for Statistical Computing, Vienna, Austria) package “metafor” was used for data analysis.^29^^,^^30^

## RESULTS

The bibliographic search yielded a total of 99 studies, of which 37 were identified as duplicates. Subsequently, 62 studies underwent a comprehensive full-text screening process. Among these, 43 studies did not meet the inclusion criteria, resulting in the inclusion of 19 eligible studies. Furthermore, through a manual search within the reference lists of relevant articles pertaining to the topic, an additional 7 studies were uncovered. Consequently, this review included 26 studies. Specifically, 12 studies investigated the causal relationship between micronutrients and COVID-19 disease, 4 studies explored the causal link between micronutrients and inflammatory markers, and 12 studies investigated the causal association between inflammatory markers and COVID-19 disease. The sum of these numbers does not equal 26, as one of the studies evaluated all these associations within the same study.^31^

Of the 26 studies included in this review, the majority considered a 2-sample MR approach (*n* = 26) and/or a 1-sample approach (*n* = 2). In addition, most studies used the HGI (Host Genetic Initiative) population as the study population source for the COVID-19 disease, although other study populations with GWAS data, such as UK Biobank (UKB) (*n* = 12), SUNLIGHT consortium (*n* = 1), Rotterdam (*n* = 1), and Interval and the Health and Retirement Study (*n* = 3), among others, were also used to carry out the study of causal association between the IV and the exposure/outcome. The exposure variables were derived from biomarkers; any study considered micronutrients from dietary data.

The results according to the exposure–outcome associations that have been addressed in this review (ie, the causal association between micronutrients and COVID-19, micronutrients and inflammatory biomarkers, and biomarkers and COVID-19) are detailed below:

### Mendelian Randomization Studies Evaluating the Micronutrient and COVID-19 Association

In total, 12 studies evaluated the causal association between different micronutrients and COVID-19 disease (**Table 2**).^31–42^ These studies comprised 8 studies focusing on VD,^31–38^ 1 study examining vitamin C,^39^ another exploring iron,^40^ and 2 additional studies^41^^,^^42^ considering more than 1 micronutrient (eg, zinc, copper, selenium, magnesium, and vitamin B_6_).

**Table 2.** Micronutrients and COVID-19: Mendelian Randomization Studies Evaluating the Causal Association Between Various Micronutrients and COVID-19 Disease

Study (year)	**Study population and sample size** ^a^	SNPs for IV	Exposure (E) and outcome (O)	MR assessment	MR study type
Au Yeung et al (2022)^31^	IV assessment: 417 580 participants Outcomes: COVID-19 I: 2 942 817 participants H: 2 401 372 participants S: 1 163 698 participants GWAS source: GWAS, HGI	IV 25(OH)D: 107 SNPs^b^	E = 25(OH)D O = COVID-19 (I; H; S)	25(OH)D → COVID-19 I (OR per 1 SD =1.00; OR Egger = 1.00) H (OR per 1 SD=1.01; OR Egger = 1.01) S (OR per 1 SD= 0.96; OR Egger = 1.00)	Two-sample MR IVW MR Egger test
Butler-Laporte et al (2021)^32^	IV assessment: 443 734 participants Outcomes: COVID-19 I: 14 134 ca/1 284 876 co H: 6406 ca/902 088 co S: 4336 ca/623 902 co GWAS source: UKB, HGI	IV 25(OH)D: 80 SNPs Secondary MR analysis: 11 SNPs involved in vitamin D pathways: Synthesis*: DHCR7/NADSYN1; CYP2R1* Transportation: *GC* Degradation*: CYP24A1*	E = 25(OH)D O = COVID-19 (I; H; S)	25(OH)D → COVID-19 I (OR per 1 SD = 0.95; *P* = .44; *P* Egger = .39) H (OR per 1 SD = 1.09; *P* = .41; *P* Egger = .81) S (OR per 1 SD = 0.97; *P* = .77; *P* Egger = .59) Secondary MR analysis: I (OR per 1 SD = 0.94; *P* = .39; *P* Egger >.05) H (OR per 1 SD = 1.04; *P* = .84; *P* Egger >.05) S (OR per 1 SD = 0.92; *P* = .59; *P* Egger >.05)	Two-sample MR IVW MR Egger test
Li et al (2021)^33^	IV assessment: 417 342 participants Outcomes: COVID-19 GWAS source: UKB	IV 25(OH)D: 134 SNPs (NA)	E = 25(OH)D O = COVID-19 (I)	25(OH)D → COVID-19 I (OR = 0.77; *P* = .16; *P* Egger = .161)	Two-sample MR IVW MR Egger test
Cui and Tian (2021)^34^	IV assessment: 417 580 participants Outcomes: COVID-19 I: 38 984 ca/1 644 784 co H: 9986 ca/1 877 672 co S: 5101 ca/1 383 241 co GWAS source: UKB, HGI	IV 25(OH)D: SNPs^b^ I: 106 SNPs H: 109 SNPs S: 109 SNPs	E = 25(OH)D O = COVID-19 (I; H; S)	25(OH)D → COVID-19 I (OR = 0.90; *P* = .047) H (OR = 1.07; *P* = .48) S (OR = 1.03; *P* = .84) MR-Egger (OR overall = 0.9404; *P* = .4834)	Two-sample MR IVW MR Egger test
Amin and Drenos (2021)^35^	IV (vitamin D levels) assessment: 488 377 participants IV (vitamin D deficiency) assessment: NA participants Outcomes: COVID-19 I: 11 181 ca/116 456 co S: 1389 ca/5879 co GWAS source: GWAS, UKB, HGI	IV 25(OH)D (levels): NA IV 25(OH)D (deficiency):17 SNPs^b^	E = 25(OH)D levels; 25(OH)D (deficiency <25 nmol/L) O = COVID-19 (I; S)	25(OH)D levels → COVID-19 I (ln OR per 1 SD = 0.17; *P* = .39; *P* Egger = .22) S (ln OR per 1 SD = 0.36; *P* = .57; *P* Egger = .43) 25(OH)D deficiency → COVID-19 I (ln OR per 1 SD = 0.04; *P* = .25; *P* Egger = .14) S (ln OR per 1 SD = 0.24; *P* = .14; *P* Egger = .20)	Two-sample MR IVW MR Egger test
Liu et al (2021)^36^	IV assessment: 417 580 participants Outcomes: COVID-19 I: 6696 ca/1 073 072 co (whole population) I negative: 3523 ca/36 634 co (test negative) S confirmed: 536 hospitalized laboratory confirmed/329 391 co (all population) S confirmed and respiratory failure: 1610 hospitalization with respiratory failure and confirmed COVID-19/2205 co (all population) GWAS source: UKB, HGI	IV 25(OH)D: 143 SNPs^b^	E = 25(OH)D O = COVID-19 (I; S)	25(OH)D → COVID-19 I (OR=1.14; *P* = .07; *P* Egger = .01) I negative (OR = 1.17; *P* = .128; *P* Egger = .044) S confirmed (OR = 0.89; *P* = .246; *P* Egger = .855) S confirmed and respiratory failure (OR = 0.89; *P* = .603; *P* Egger = .682)	Two-sample MR IVW MR Egger test
Patchen et al (2021)^37^	IV-1/IV-2/IV-3 assessment (serum vitamin D): 401 460 participants IV-4 assessment (vitamin D deficiency/insufficiency): 16 905 participants Outcomes: COVID-19 GWAS source: UKB, HGI	IV 25(OH)D: NA SNPs IV-1 (vitamin D transport and metabolism): *GC*, *CYP2R1*, *DHCR7, CYP24A1* IV-2 (vitamin D metabolism): *GC, CYP2R1, DHCR7, CYP24A1, SEC23A, AMDHD1* IV-3 (expanded instrument: 63 additional loci associated with serum vitamin D) IV-4 (vitamin D deficiency and insufficiency): *GC, DHCR7, CYP2R1*	E = 25(OH)D levels, 25(OH)D deficiency O = COVID-19 (I; H; S)	25(OH)D levels → COVID-19 IV-1 (*P*: NA) COVID vs Populat OR per SD = 1.04; *P* > .05 Hospit COVID vs Populat OR =1.05; *P* > .05 Sev Resp COVID vs Populat OR = 0.96 COVID vs COVID Neg OR = 1.15 Hospit vs NonHospit COVID OR = 1.44 IV-2 (*P*: NA) COVID vs. Populat OR = 1.04 Hospit COVID vs Populat OR = 1.15 Sev Resp COVID vs Populat OR = 1.01 COVID vs COVID Neg OR = 1.15 Hospit vs NonHospit COVID OR = 1.42 IV-3 (*P*: NA) COVID vs Populat OR = 1.02 Hospit COVID vs Populat OR = 1.12 Sev Resp COVID vs Populat OR = 1.05 COVID vs COVID Neg OR = 1.05 Hospit vs NonHospit COVID OR = 1.23 IV-4 deficiency/insufficiency (*P*: NA) COVID vs Populat OR = 1.00 Hospit COVID vs Populat OR = 1.00 Sev Resp COVID vs Populat OR = 1.02 COVID vs COVIDNeg OR = 0.99 Hospit vs NonHospit COVID OR = 0.97 MR-Egger, overall: COVID vs Populat, *P* = .32 Hospit COVID vs Populat, *P* = .62 Sev Resp COVID vs Populat, *P* = .89 COVID vs COVID Neg, *P* = .17 Hospit vs NonHospit COVID, *P* = .92	Two-sample MR IVW MR Egger test
Qiu et al (2023)^38^	IV assessment: 417 580 participants Outcomes: COVID‐19 38 984 ca/1 644 784 co GWAS source: UKB, HGI	IV 25(OH)D: 85 SNPs^b^	E = 25(OH)D O = COVID-19	25(OH)D → COVID-19 OR= 1.03; *P* = .54, *P* Egger = .995	Two-sample MR IVW MR Egger test GSMR 100
Hui et al (2021)^39^	IV assessment: 52 018 participants Outcomes: COVID-19 I: 29 071 ca/1 559 712 co H: 9373 ca/1 197 256 co S: 4606 ca/702 801 co GWAS source: GWAS, HGI	IV vitamin C: 9 SNPs (rs2559850; rs117885456; rs10136000; rs56738967; rs9895661; rs6693447; rs13028225; rs33972313; rs10051765)	E = vitamin C O = COVID-19 (I; H; S)	Vitamin C → COVID-19 I (OR = 1.04; *P* = .51; *P* Egger = .61) H (OR = 1.10; *P* = .35; *P* Egger = .81) S (OR = 1.00; *P* = .99; *P* Egger = .70)	Two-sample MR IVW MR Egger test
Mohus et al (2022)^40^	IV assessment: 246 139 participants Outcomes: COVID-19 H: 4829 ca/11 816 co GWAS source: UKB, HGI	IV (iron [Fe] and related markers): SNPs^b^	E = iron, TSAT, TIBC, ferritin O = COVID-19 (H)	Iron → COVID-19 H1 (OR = 1.29; *P* = .08; *P* Egger = .51) TSAT → COVID-19 H1 (OR = 1.29; *P* = .17; *P* Egger = .99) TIBC → COVID-19 H1 (OR = 1.0; *P* = .99; *P* Egger = .60) Ferritin → COVID-19 H1 (OR = 1.15; *P* = .36; *P* Egger = .79)	Two-sample MR IVW MR Egger test
Sobczyk and Gaunt (2022)^41^	IV assessment: NA GWAS source: Open GWAS, GWAS catalog, HGI	IV zinc (Zn): 2 SNPs (rs2120019; rs1532423) IV selenium (Se): 2 SNPs (rs921943; rs6859667) IV copper (Cu): 2 SNPs (rs1175550; rs2769264) IV vitamin K_1_: 3 SNPs (rs4645543; rs4852146; rs6862071)	E = Zn, Se, Cu, vitamin K_1_ O = COVID-19 (I; H; S)	Zn → COVID-19 I (OR per 1 SD = 0.97; *P* = .55) H (OR per 1 SD = 1.06; *P* = .66) S (OR per 1 SD=1.21; p=0.39) Se → COVID-19 I (OR per 1 SD = 1.03; *P* = .5) H (OR per 1 SD = 0.98; *P* = .71) S (OR per 1 SD = 0.99; *P* = .86) Cu → COVID-19 I (OR per 1 SD = 1.07; *P* = .06) H (OR per 1 SD = 1.07; *P* = .49) S (OR per 1 SD = 1.13; *P* = .4) Vitamin K_1_ → COVID-19 OR per 1 SD = NA; *P* = nonsignificant	Two-sample MR IVW MR
Daniel et al (2022)^42^	IV assessment: NA participants Outcomes: COVID-19 I: 87 870 ca/2 210 804 co GWAS source: GWAS catalog, PubMed, HGI	IV vitamin B_6_: 2 SNPs (rs4654748; rs1256335) IV magnesium (Mg): 3 SNPs (rs13146355; rs11144134; rs3925584)	E = vitamin B_6_; Mg; others micronutrients O = COVID-19 (I)	Vitamin B_6_ → COVID-19 I (OR = 1.06; *P* = .036; *P* Egger = NA) Mg → COVID-19 I (OR = 0.33; *P* = .042; *P* Egger = .208) Others micronutrients → COVID-19 No significant associations were found	Two-sample MR IVW MR Egger test

Results shown in tables are those reported by the study using the IVW method unless stated otherwise. *P* values corresponding to the MR-Egger test (the intercept) are indicated to show potential pleiotropy in the results.

a The study population is the number of participants available for the IV assessment.

b List of SNPs reported in the article’s supplementary material.

Abbreviations: ca, cases; co, controls; GSMR, generalized summary-data-based Mendelian randomization; GWAS, genome-wide association study; H, hospitalization; HGI, Host Genetics Initiative; Hospit, hospitalized; I, infection; IV, instrumental variable; IVW, inverse variance weighted; ln, log normal; MR, Mendelian randomization; NA, not available; NonHospit, non-hospitalized; OR, odds ratio; Populat, population; Resp, respiratory; Sev, severe; SNP, single nucleotide polymorphism; UKB, UK Biobank; S, severity; TIBC, total iron binding capacity; TSAT, transferrin saturation; 25(OH)D, 25-hydroxyvitamin D.

Overall, of the 8 studies that evaluated the causal association between VD and COVID-19, none reported significant associations between this nutrient and the disease. Similarly, the studies by Hui et al^39^ and Mohus et al^40^ on the causal association between vitamin C or iron and COVID-19 disease, respectively, did not find any significant associations. Among the 2 studies that evaluated multiple micronutrients, only the study by Daniel et al^42^ found a significant association between vitamin B_6_ (OR = 1.03) and magnesium (OR = 0.33) with the risk of infection, but not for other micronutrients (calcium, copper, iron, phosphorus, selenium, zinc, β-carotene, VD, and vitamin B_12_).

Of all the studies, only 3 evaluated the association between micronutrients and different aspects of COVID-19, including infection, hospitalization, and disease severity.^33^^,^^38^^,^^42^ With regard to infection, the above-mentioned study by Daniel et al^42^ was the only one that reported significant findings for magnesium and vitamin B_6_. However, none of the 9 studies examining hospitalization and disease severity stated significant results. Among the 3 studies that did not account for disease states, 2 studies^33^^,^^42^ evaluated the risk of COVID-19 infection, with one of them yielding significant results,^42^ whereas in the third study,^38^ a causal association with COVID-19 disease in general was evaluated, although nonsignificant results were obtained.

All studies applied a 2-sample MR approach, with all but 1 study using the inverse variance weighted method to derive causal estimates.^33^ It is noteworthy that, while the study by Li et al^33^ stated a 2-sample MR approach, it could be categorized as a 1-sample MR study given that the 2 study populations (UKB and UK10K project) were used to cross-validate the results. The ancestry of the study populations, from which the genetic variants were derived, was of European descent in all studies. Additionally, all studies explored the presence of pleiotropy via the MR-Egger method. Only one of these studies^36^ showed a correlation between the IV and the COVID-19 infection trait (Egger *P* = .013).

Varying numbers of genetic variants were considered for the IV built in each study, with over 80 single nucleotide polymorphisms (SNPs) included in VD-related IVs, and IVs accounting for several genes related to VD metabolism (*GC, CYP24A1, CYP2R1, SEC23A*, etc) being considered in 2 studies.^32^^,^^37^ Of note, one of these studies^37^ and an additional study^35^ accounted for genetic variants associated with VD deficiency as an IV. In addition, the study by Butler-Laporte et al^32^ performed a secondary MR analysis by focusing on certain VD pathways. As mentioned above, none of these studies yielded a significant association with COVID-19 risk or disease outcomes.

As shown in **Tables S1**^14^^,^^31–55^ and **S2**^14^^,^^31–55^ on the risk-of-bias assessment, the methodological quality of these studies was high in 4 studies^34^^,^^38^^,^^40^^,^^42^ but low in the remaining studies.^31–33^^,^^35–37^^,^^39^^,^^41^ The studies by Daniel et al^42^ and Cui and Tian^34^ reached the highest scores (>22 points).

**Figure 3.**
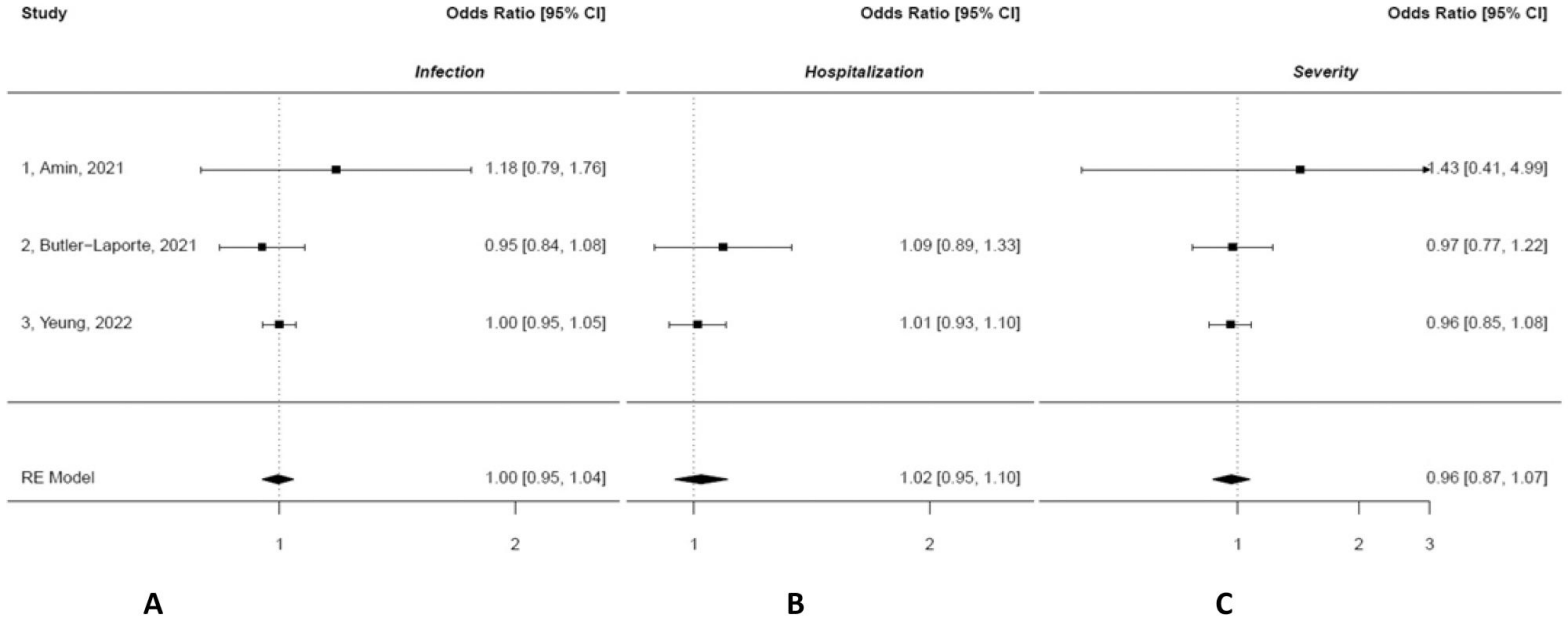
Meta-analyses of Studies Reporting Results on Vitamin D and COVID-19 Disease Outcomes. Odds ratios, 95% CIs of each study, and combined MR estimates are shown. (A) Infection. (B) Hospitalization. (C) Severity. Estimates of those studies without study population overlap at the exposure level were pooled.^31^^,^^32^^,^^35^ Abbreviation: RE, Random Effects

Figure 3 ^31^ ^,^ ^32^ ^,^ ^35^ shows the results of the meta-analyses on VD and COVID-19 by disease outcome (infection, hospitalization, and severity). Pooled estimates confirmed that VD was not significantly associated with any outcome (*P* > .05). There was no evidence of heterogeneity or publication bias in these analyses. Random- and fixed-effects meta-analyses yielded similar results (data not shown).

### Mendelian Randomization Studies Evaluating the Association Between Micronutrients and Biomarkers of Inflammation

A total of 4 studies evaluated the possible causal association between VD and various inflammatory markers, without considering other micronutrients (Table 3^14^^,^^31^^,^^43^^,^^44^). The most commonly assessed inflammatory marker as an outcome variable was CRP, with 3 studies exploring the connection between VD and this marker.^14^^,^^43^^,^^44^ In one of the studies,^43^ in addition to CRP, the association with other inflammatory markers, such as soluble intercellular adhesion molecule-1 (sICAM1) and alpha 1-glycoprotein (AGP), was also evaluated. The ACE marker was considered in the study by Au Yeung et al.^31^ However, none of the studies reported significant findings on the causal association between VD and CRP, nor did they find significant associations with the other inflammatory markers. Furthermore, bidirectional MR analyses also yielded nonsignificant results. Interestingly, the association between VD and CRP was found to be nonlinear and statistically significant for VD levels below 25 nmol/L. Pooled estimates between the latter and other studies on VD and CRP could not be derived due to variations in results reporting and overlap of study populations.

**Table 3.** Micronutrients and Inflammatory Biomarkers: Mendelian Randomization Studies Evaluating the Causal Association Between Various Micronutrients and Inflammatory Biomarkers

Study (year)	**Study population and sample size** ^a^	SNPs for IV	Exposure (E) and outcome (O)	MR assessment	MR study type
Zhou and Hyppönen (2022)^14^	IV assessment: 294 970 participants GWAS source: UKB, SUNLIGHT Consortium	IV-1 25(OH)D: 35 SNPs^b^ Sensitivity analysis for 21 SNPs^b^: *GC, DHCR7, CYP2R1*, and *CYP24A1* IV-2 (CRP): 46 SNPs^b^	E = 25(OH)D O = CRP	25(OH)D → CRP Nonlinear MR (*P* = 1.49 × 10^−4^); inverse association for <25 nmol/L 25(OH)D *P* Egger = NA CRP → 25(OH)D Linear MR (*P* = .32) Nonlinear MR (*P* = .76) *P* Egger = NA	One- and 2-sample MR GRS and IVW MR, respectively Linear and nonlinear MR Bidirectional MR Egger test
Au Yeung et al (2022)^31^	IV assessment: 417 580 participants GWAS source: GWAS studies, HGI	IV 25(OH)D: 107 SNPs^b^	E = 25(OH)D O = ACE2	25(OH)D → ACE2 β per SD = –0.06; *P* Egger >.05	Two-sample MR IVW MR Egger test
Palaniswamy et al (2020)^43^	IV assessment: 337 199 participants GWAS source: GWAS, UKB	IV 25(OH)D: 25 SNPs^b^	E = 25(OH)D O = CRP, AGP, sICAM-1	25(OH)D → markers CRP (*P* = .686; *P* Egger = .614) sICAM-1 (*P* = .585; *P* Egger = .691) AGP (*P* = .748; *P* Egger = .054)	Two-sample MR IVW MR Egger test
Liefaard et al (2015)^44^	IV assessment: 14 926 participants GWAS source: Rotterdam Study	IV-1 25(OH)D: 4 SNPs (rs12785878; rs10741657; rs2282679; rs6013897) IV-2 (CRP): 18 SNPs (rs2794520; rs4420638; rs1183910; rs4420065; rs4129267; rs1260326; rs12239046; rs6734238; rs9987289; rs10745954; rs1800961; rs340029; rs10521222; rs12037222; rs13233571; rs2847281; rs6901250; rs4705952)	E = 25(OH)D O = CRP	25(OH)D → CRP (β = –0.018, *P* = .082) CRP → 25(OH)D (β = 0.001, *P* = .998)	One-sample MR GRS MR Bidirectional MR

a The study population is the number of participants available for the IV assessment.

b List of SNPs reported in the article’s supplementary material. The results shown in the tables are those reported by the study using the IVW method. *P* values corresponding to the MR–Egger test are indicated to show potential pleiotropy in the results.

Abbreviations: ACE2, angiotensin converting enzyme 2; AGP, alpha 1-glycoprotein; CRP, C-reactive protein; GRS, genetic risk score; HGI, Host Genetics Initiative; IV, instrumental variable; IVW, inverse variance weighted; MR, Mendelian randomization; UKB, UK Biobank; GWAS, genome-wide association study; sICAM-1, soluble intercellular adhesion molecule-1; SNP, single nucleotide polymorphism; 25(OH)D, 25-hydroxyvitamin D.

Three of the 4 included studies^14^^,^^31^^,^^43^ were based on a 2-sample MR approach, whereas the remaining study used a single study sample.^44^ Among the studies, the study populations were diverse, including the UKB, various GWASs, the SUNLIGHT Consortium, and the Rotterdam Study, all of European ancestry. Two of the studies, the one carried out by Liefaard et al^44^ and that of Zhou and Hyppönen,^14^ performed bidirectional MR studies, enabling the assessment of reverse causation between both micronutrients and inflammatory marker associations. Potential pleiotropic effects by the Egger test were evaluated in 3 studies.^14^^,^^31^^,^^43^ The number of SNPs used to build the IV associated with VD levels [25(OH)D] varied between the studies from 4^44^ to 107 SNPs,^31^ with genetic variants from multiple genes being included (eg, *GC*, *CYP2R1*, *CYP24A1*, *DHCR7*).

With regard to the risk of bias (**Tables S1** and **S2**),^14^^,^^31–55^ overall, the methodological quality of these studies was low. The studies by Zhou and Hyppönen^14^ and Palaniswamy et al^43^ reached the highest scores (>22 points).

### Mendelian Randomization Studies Evaluating the Inflammation-Related Markers–COVID-19 Association

A total of 12 studies evaluated the possible causal association between different inflammatory-related markers and COVID-19 disease (Table 4^31^^,^^45–55^). These studies comprised the following inflammatory-related markers: proteins in 5 studies^45–49^; ACE in 5 studies^31^^,^^48–51^; kidney and liver function markers in 1 study^52^; cytokines in 1 study^53^; metabolic syndrome–related markers including adiposity, blood pressure, and glycemic traits in 1 study^31^; and other cardiovascular risk factor–related markers including lipoproteins in 2 studies.^54^^,^^55^

**Table 4.** Inflammatory Biomarkers and COVID-19: Mendelian Randomization Studies Evaluating the Causal Association Between Various Inflammatory Biomarkers and COVID-19

Study (year)	**Study population and sample size** ^a^	SNPs for IV	Exposure (E) and outcome (O)	MR assessment	MR study type
Au Yeung et al (2022)^31^	IV assessment: 28 204 participants Outcomes: COVID-19 I: 2 942 817 participants H: 2 401 372 participants S: 1 163 698 participants GWAS source: GWAS, HGI	IV (ACE2): 3 SNPs (rs1849863; rs143380244; rs73202884)	E = ACE2 O = COVID-19 (I, H, S)	ACE2 → COVID-19 I (OR per SD = 1.11; *P* < .05) H (OR per SD = 1.21; = *P* < .05) S (OR per SD = 1.21; *P* < .05)	Two-sample IVW MR
Kousathanas et al (2022)^45^	IV assessment: NA participants Protein-like IVs Outcomes: COVID-19 GWAS source: Interval study Health for proteins, Retirement Study for COVID-19	IV (proteins): 16 genes from transcriptome analysis (gene expression) *ICAM5*; *GOLM1*; *ICAM1*; *FAM3D*; *PDGFRL*; *CD209*; *ABO*; *CIGALT1C1*; *CCL25*; *F8*; *TLR4*: *LY96*; *IL3RA*; *SELE*; *CAMK1*; *IL27RA*	E = proteins O = COVID-19	Protein levels → COVID-19 ICAM5: β = -0.07, *P* = 7.65 × 10^−8^ GOLM1: β = 0.20, *P* = 1.04 × 10^−21^ ICAM5: β = -0.048, *P* = .0054 FAM3D: β = 0.12, *P* = 3.12 × 10^−18^ PDGFRL: β = 0.021, *P* = .041 CD209: β = 0.11, *P* = 1.88 × 10^−15^ *ABO*: β = 0.084, *P* = 7.76 × 10^−22^ F8: β = 0.16, *P* = 1.46 × 10^−14^ IL3RA: β = -0.065, *P* = 4.33 × 10^−6^ SELE: β = -0.095, *P* = 3.76 × 10^−14^ *P <* .05*: ICAM1, CIGALT1C1, CCL25, CAMK1, IL27RA	Two-sample GSMR 100
Richardson et al (2021)^46^	IV assessment: NA participants Protein-like IVs Outcomes: COVID-19 S GWAS source: Fenland population-based cohort study, UKB, HGI	IV (proteins): 97 pQTLs associated with COVID-19 risk factors BMI; SBP; DBP; HDL; LDL; TG; apo A-I and B; smoking, waist-hip ratio, childhood adiposity 18 pQTLs, weakly correlated with each other (rs66908049; rs3842913; rs10070449; rs189547345; rs254987; rs62361958; rs77847765; rs7729009; rs6875155; rs6450360; rs9292108; rs62363942; rs13166579; rs13182056; rs75187300; rs111296580; rs13162507; rs188841)	E = COVID-19–related proteins O = COVID-19	COVID-19 risk factors → proteins: Across the 11 exposures, there were 97 genetically predicted effects on circulating proteins. Significant finding for: Gp130 (OR = 1.81; *P* = .002) Protein levels → COVID-19 On the 18 pQTLs-based IV: S (OR Egger = 1.55; *P* Egger = .34)	Two-sample IVW MR Egger test
Gaziano et al (2021)^47^	IV assessment: >1 million participants Protein-like IVs Outcome: COVID-19 H 7554 ca GWAS source: Fenland and Interval study for proteins, HGI and MVP for COVID-19	IV (proteins): pQTLs (plasma) of 1263 actionable proteins eQTLs (tissue) of 1265 actionable proteins Instruments of eQTLs were significant and assessed as IV	E = COVID-19–related proteins O = COVID-19 (H)	Protein levels → COVID-19 MR results for 6 genes (*IL10RB*, *CCR1*, *IFNAR2*, *PDE4A*, *ACE2*, and *CCR5*) in at least 1 tissue were significant; *P* < 3.96 × 10^−5^; *P* Egger = NA or <.05, for some variants (in instrument of *IL10RB* and *PDE4A*)	Two-sample IVW MR Egger test
Zheng (2022)^48^	IV assessment: NA participants Protein-like IVs Outcomes: COVID-19 I (COVID-19 vs negative): 9637 ca/106 138 co I (COVID-19 vs population): 14 134 ca/1 284 876 co I (COVID-19 vs population): 17 965 ca/1 370 547 co S (hospitalized vs population): 7885 ca/9 61 804 co GWAS source: GWAS catalog, HGI	IV (ACE): *cis*-eQTLs related to ACE2 expression and ACE2 levels 21 Significant SNPs associated with plasma ACE2^b^	E = ACE O = COVID-19 (I, S)	ACE → COVID-19 MR estimates of ACE2 were positive for both I and S of COVID-19 OR: NA	Two-sample IVW MR
Yang et al (2022)^49^	IV assessment: NA participants Protein-like IVs Outcomes: COVID-19 I; H; S GWAS source: public data for proteins and ORIGIN trial, HGI within GenOMICC consortium for COVID-19	IV (ACE2): *cis*-pQTLs and *trans*-pQTLs of SNPs related to ACE2 expression and ACE2 levels 10 Significant SNPs associated with plasma ACE2 (rs3094087, rs2954021, rs1169288, rs28929474, rs2274685, rs340005, rs17616063, rs1800961, rs5992134, rs1849863)	E = ACE2 O = COVID-19 (I, H, S)	ACE2 expression → COVID-19 I (OR = 1.60; *P* = .02) H (OR =1.52; *P* = .03) S (OR =1.63; *P* = .01)	Two-sample IVW MR and GSMR 100
Butler-Laporte et al (2022)^50^	IV assessment: 4147 (ORIGIN trial) participants 3200 (AGES cohort) participants Outcome: COVID-19 I: (A) 3382 ca/37 851 co; (B) 6182 ca/960 186 co H: (A) 677 ca/2372 co; (B) 2710 ca/813 243 co S: (A) 213 ca/750 co; (B) 540 ca/366 840 co GWAS source: ORIGIN trial, AGES cohort, HGI	IV (ACE): 12 SNPs (rs4343; rs1074637; rs11650201; rs12452187; rs12602457; rs13342595; rs2137143; rs4968780; rs72847305; rs74251225; rs75457471; rs79480822)	E = ACE O = COVID-19 (I, H, S)	ACE → COVID-19 (ORIGIN trial + HGI) I: (A) OR per SD = 1.02; *P* = .76; *P* Egger = .39; (B) OR per SD = 1.03; *P* = .48; *P* Egger = .52 H: (A) OR per SD = 0.86; *P* = .20; *P* Egger = .78; (B) OR per SD = 0.94; *P* Egger = .71 S: (A) OR per SD = 0.74; *P* = .10; *P* Egger = 0.84; (B) OR per SD = 0.92; *P* Egger = .86 ACE → COVID-19 (AGES cohort + HGI) I (OR per SD = 0.98, *P* = .76) H (OR per SD = 0.86, *P* = .23) S (OR per SD = 0.75, *P* = .18)	Two-sample IVW MR Egger test
Gill et al (2020)^51^	IV assessment: 4947 participants Outcomes: COVID-19 hospitalization COVID-19: 6492 ca/1 012 809 co GWAS source: Interval, HGI	IV (ACE2): 17 SNPs SNPs = NA	E = ACE2 O = COVID-19	ACE → COVID-19 H: OR per SD = 1.02; *P* > .05; *P* Egger = NA	Two-sample IVW MR
Sood et al (2023)^52^	IV assessment: 4147 participants with cardiovascular risk factors Outcomes: COVID-19 hospitalized: 5773 ca/15 497 co GWAS source: ORIGIN, HGI	IV: Several SNPs associated with 15 inflammation markers related to COVID-19 hospitalization SNPs = NA	E = inflammation markers O = COVID-19 Mediators = BMI	Biomarker → COVID-19 KIM-1—COVID-19 H: OR = 0.86; *P* = 3.81 × 10^−4^; *P* Egger = .14 Mediation analysis: KIM-1 mediated the association BMI–COVID-19 H OR = 1.23; *P* = 5.65 × 10^−3^	Two-sample IVW MR
Li et al (2021)^53^	IV assessment: 8293 participants Outcomes: COVID-19 S: 36 590 ca/1 668 938 co H: 12 888 ca/1 295 966 co GWAS: FINRISK, HGI	IV: Several SNPs associated with 41^b^ inflammation markers related to COVID-19 SNPs = NA	E = inflammation markers O = COVID-19 41 Plasma/serum cytokines	Cytokines → COVID-19 MIP1b—COVID-19: S: OR per SD = 0.92; *P* < .05* H: OR per SD = 0.93; *P* < .05* IL-6 and others: S and H: *P* > .05 COVID-19 → cytokines Inverse associations: *P* < .05* GCSF, HGF, IL2RA, MCSF, TNFb, TRAIL *P* Egger = NA	Two-sample IVW MR Bidirectional
Wang et al (2021)^54^	IV assessment: 361 194 participants Outcomes: COVID-19 S: 6492 ca/1 012 809 co H: 12 888 ca/1 295 966 co GWAS source: UKB, HGI	IV: Several SNPs associated with 9 inflammation markers (albumin, Ibil and Tbil, total protein, AST, ALT, ALP, CGT, creatinine, WBC) related to COVID-19 SNPs = NA	E = inflammation markers O = COVID-19	Albumin → COVID-19 S: OR = 0.85; *P* = .024* Bilirubin → COVID-19 S: OR = 1.10; *P* = .023* Other → COVID-19 *P* > .05	Two-sample IVW MR
Zhu et al (2021)^55^	IV assessment: 466 participants Outcomes COVID-19 severity: Mild: 6 Moderate: 164 Severe: 227 Critical: 69 GWAS source: GWAS catalog, HGI	IV: Several SNPs associated with 7 inflammation markers (apoA, APTT, Ibil and Tbil, LDL, LpA, WBC) related to COVID-19 SNPs apoA: rs11032789 APTT: rs1801020 Ibil: rs28946889 LDL: rs7412 LpA: rs56393506 Tbil: rs28946889 WBC: rs9268517	E = inflammatory markers O = COVID-19	One-sample: Any marker → COVID-19 severity *P* > .05 Two-sample: Any marker → COVID-19 severity *P* > .05	One-sample and 2-sample Wald ratio and IVW MR, respectively

* Significant *P* value.

a The study population is the number of participants available for the IV assessment.

b List of SNPs reported in the article’s supplementary material; I (infection risk); H (hospitalized); S (severity).

Abbreviations: ABO, ABO glycosyltransferase gene; ACE2, angiotensin converting enzyme 2; AGES cohort, AGES Reykjavik study; apoA, apolipoprotein A; ALP, Alkaline Phosphatase; ALT, Alanine aminotransferase; APTT, activated partial thromboplastin time; AST, Aspartate aminotransferase; BMI, body mass index; ca, cases; CAMK1, calcium/calmodulin dependent protein kinase 1; CCL25, C-C motif chemokine ligand 25; CD209, CD209 molecule; CGT, Cerebroside Sulfotransferase; co, controls; DBP, diastolic blood pressure; eQTL, expression quantitative trait locus; F8, coagulation factor VIII; FAM3D, FAM3 metabolism regulating signaling molecule D; FINRISK, The National FINRISK Study; GCSF, granulocyte colony-stimulating factor; GOLM1, Golgi membrane protein 1; Gp130, glycoprotein 130; GSMR, generalized summary-data-based Mendelian randomization; GWAS, genome-wide association study; HDL, high-density-lipoprotein cholesterol; HGF, hepatocyte growth factor; HGI, Host Genetic Initiative; Ibil, indirect bilirubin; ICAM5, intercellular adhesion molecule 5; ICAM1, intercellular adhesion molecule 1; IL3RA, interleukin 3 receptor subunit alpha; IL2RA, IL-2 receptor alpha subunit; IL27RA, interleukin 27 receptor subunit alpha; IV, instrumental variable; KIM-1, hepatitis A virus cellular receptor 1 (also known as kidney injury molecule-1); LDL, low-density-lipoprotein cholesterol; LpA, lipoprotein A; MCSF, macrophage colony-stimulating factor; MIP1b, macrophage inflammatory protein-1β; MR, Mendelian randomization; MVP, Million Veteran Program; NA, not available; OR, odds ratio; ORIGIN, Outcome Reduction with Initial Glargine INtervention; PDGFRL, platelet-derived growth factor receptor like; pQRL, protein quantitative trait locus; SBP, systolic blood pressure; SELE, selectin E; SNP, single nucleotide polymorphism; Tbil, total bilirubin; TG, triglycerides; TNFb, tumor necoris factor-beta; TRAIL, TNF-related apoptosis inducing ligand; UKB, UK Biobank; WBC, white blood cell count.

The 5 studies that evaluated the association between circulating inflammatory proteins and COVID-19^45–49^ were all based on gene expression data, considering either protein quantitative trait locuses (pQTLs) in plasma,^46–49^ expression QTLs (eQTLs) in tissue, or both.^47^ Thus, the IV considered in these studies was made of SNPs that were related to genes encoding proteins that were found to be relevant in COVID-19 disease in previous studies.

Only the study by Kousathanas et al^45^ found some significant associations between certain proteins and COVID-19, such as ICAM5 (Intercellular Adhesion Molecule 5 gene), GOLM1 (Golgi membrane protein 1), ICAM5, FAM3D (Family with Sequence Similarity 3, Member D), PDGFR (Platelet-Derived Growth Factor Receptor), CD209 (Cluster of Differentiation 209), ABO (ABO Blood Group), F8 (Coagulation Factor VIII), IL3RA (Interleukin 3 Receptor Alpha), and SELE (Selectin E). The SNPs of genes encoding proteins such as IL10RB (Interleukin 10 Receptor Subunit Beta), CCR1 (C-C Chemokine Receptor Type 1), IF-NAR2 (Interferon Alpha and Beta Receptor Subunit 2), PDE4A (Phosphodiesterase 4A), ACE2 (Angiotensin I Converting Enzyme 2), and CCR5 (C-C Chemokine Receptor Type 5) were also found to be associated with COVID-19 in the study by Gaziano et al^47^, as well as those encoding the protein Gp130^46^ or ACE2 expression.^49^ Associations by disease outcomes were only evaluated in the study by Yang et al^49^ and the study by Zheng et al,^48^ where significant associations were observed for infection, hospitalization, and/or severity.

**Figure 4.**
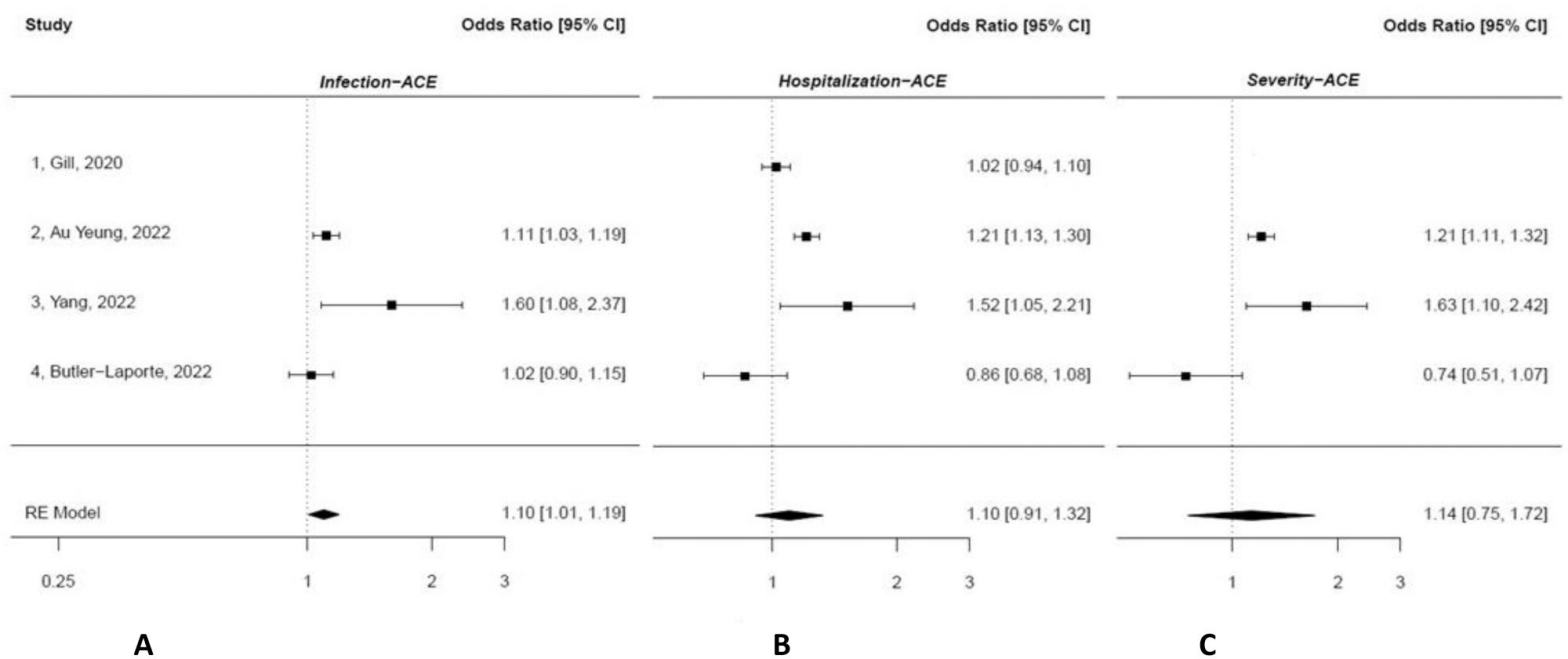
Meta-analyses of Studies Reporting Results on angiotensin-converting enzyme (ACE) 2 (ACE2) and COVID-19 Disease Outcomes. Odds ratios (ORs), 95% CIs of each study, and combined Mendelian randomization estimates are shown. (A) Infection. (B) Hospitalization. (C) Severity. There was no evidence of heterogeneity or publication bias in these analyses. Random- and fixed-effects meta-analyses yielded similar results. Estimates of those studies without study population overlap at the exposure level were pooled.^31^^,^^49–51^ Estimates were given per SD increase, except in the study by Yang et al,^49^ However, in this study, the results reported per 1-unit increase align with the SD increment. As reported in this study, overall, the SD of ACE2 is 1.17, which results in an OR = 1.43 (1.04–1.98) derived from β = log(estimate)/SD. Abbreviation: RE, Random Effects

Five studies were identified that specifically investigated associations between ACE2 and COVID-19 using IVs composed of SNPs from the ACE2 gene and its expression.^31^^,^^48–51^ Importantly, these studies were distinguished by disease outcomes. Three of these studies showed that increasing levels of ACE were positively and significantly associated with COVID-19,^31^^,^^48^^,^^49^ with the risk of infection, hospitalization, and severity increasing by 11%–63%. The study by Yang et al^49^ considered not only pQTLs related to ACE2 expression but also SNPs associated with plasma levels of ACE2. The summary statistics were derived from the ORIGIN (Outcome Reduction with Initial Glargine INtervention) trial and the HGI consortium,^49^ whereas GWAS public data and that of HGI were used in the other 2 studies.^31^^,^^48^ Conversely, the association between ACE2 and COVID-19 was not supported by 2 of the studies.^50^^,^^51^ Their results were based on the ORIGIN, AGES (AGES Reykjavik study), Interval, and HGI study populations. By pooling the results of those studies that assessed associations between IV and ACE2 in different study populations, as shown in the meta-analyses displayed in Figure 4,^31^^,^^49–51^ a significant and positive cause-and-effect relationship was found between ACE2 and COVID-19 infection (MR OR = 1.10; 95% CI: 1.01–1.19). However, no significant causal association was observed for COVID-19 hospitalization or severity.

With respect to other inflammatory markers, such as cytokines, kidney and function markers, or others related to metabolic syndrome and cardiovascular disease risk (bilirubin, Apo, AST, ALT, ALP, CGT, creatinine, etc), the following results were reported.

The study by Li et al^53^ assessed the causal association between 41 cytokines and COVID-19, with only the relationship between MIP1b (macrophage inflammatory protein-1β) and COVID-19 proving significant. In another study evaluating 15 inflammatory markers associated with COVID-19 (out of 235 markers), only KIM-1 (kidney injury molecule 1) was found to be causally linked to COVID-19 hospitalization. According to this study, this marker also mediated the association between body mass index (BMI) and COVID-19 in a statistically significant manner (Sood et al^52^). Both KIM-1 and MIP1b were inversely associated with COVID-19. Renal and hepatic biomarkers, such albumin and bilirubin, were also significantly associated with the severity of the disease.^54^ Increasing levels of albumin were associated with a decreased COVID-19 risk, whereas bilirubin was positively associated with COVID-19 risk in this study. Conversely, other inflammatory markers, including apolipoprotein A (apoA), activated partial thromboplastin time (APTT), indirect bilirubin (Ibil), total bilirubin (Tbil), low-density-lipoprotein (LDL) cholesterol, lipoprotein A (LpA), and white blood cell count (WBC), were not found to have a causal association with COVID-19 in the study conducted by Zhu et al.^55^ Pooling the results of these studies was not possible due to the wide variety of inflammatory markers considered. Summary statistics in these studies were obtained from the GWAS catalog, FINRISK, Origin, and HGI.

All the studies used a 2-sample MR approach. Only the study by Li et al^53^ carried out a bidirectional MR, whereby it was shown that the association goes in both directions. Some studies^46^^,^^47^^,^^50^ evaluated pleiotropy using the MR-Egger method. Potential pleiotropy was found only in the study by Gaziano et al.^47^ With regard to the IVs, those that were based on the expression of proteins were diverse and made up of different numbers of loci and SNPs. The ACE2 IVs comprised 3 to 21 SNPs of the ACE2 gene or SNPs associated with the expression of this gene. The IVs also varied largely between the other inflammatory marker studies.

According to the risk-of-bias assessment (**Tables S1** and **S2**),^14^^,^^31–55^ the methodological quality of these studies was high in 4 studies^50^^,^^53–55^ but low in the remaining studies.^31^^,^^45–49^^,^^51^^,^^52^ The studies by Butler-Laporte et al^50^ and Wang et al^54^ reached the highest scores (>22 points).

## DISCUSSION

This study evaluates the existing evidence concerning the causal relationship between VD, other micronutrients, and COVID-19. Various disease outcomes, including infection, hospitalization, and severity, were examined. Additionally, the impact of these micronutrients on COVID-19 inflammation-related markers was explored. While the majority of the identified studies (*n* = 8) focused on VD, a limited number (*n* = 4) investigated the roles of other vitamins and minerals (calcium, copper, iron, phosphorus, selenium, zinc, β-carotene, and vitamin B_12_) in the disease. Notably, VD was the only micronutrient for which associations with inflammatory markers were assessed. Overall, the MR studies included in this review do not support a causal association between VD and COVID-19 outcomes or related inflammation markers, except in the case of VD deficiency and CRP. In particular, VD levels below 25 nmol/L were related to increased CRP levels in an MR study that evaluated the shape of the dose–response relationship between VD and CRP,^14^ but no other MR studies investigating the linearity of the association between VD and inflammatory markers or COVID-19 outcomes were identified. The only study evaluating VD deficiency through an IV with regard to COVID-19 disease also did not support an association between VD and COVID-19.^37^ It is important to consider that, while MR studies do not support a causal association between VD and COVID-19, the high variability in study design and in the assessment of the IV, may hinder drawing any valid conclusion.

With regard to MR association studies that assessed inflammation markers and COVID-19 outcomes, of the 12 identified studies, there were studies examining either protein-related inflammatory markers, ACE2 expression, or other markers related to inflammation and metabolic syndrome/cardiovascular disease risk factors. Some causal associations were reported in these studies between COVID-19 and over 15 proteins, as well as the markers albumin, bilirubin, MPK-1, MIP1b, and ACE2. Importantly, meta-analyses involving 4 MR studies on ACE2 and COVID-19 outcomes confirmed that elevated plasma levels of ACE2 increase the risk of COVID-19 infection. However, no causal associations were observed for hospitalization and disease severity. These results suggest that, while VD is not causally associated with COVID-19 disease, an indirect link may exist through inflammation (eg, via CRP or ACE2) mediating the VD and COVID-19 relationship.

Vitamin D, a fat-soluble compound, exhibits significant variability in dietary absorption among individuals. This variability is due to several factors, including the molecular form of VD (vitamin D_3_ sourced from animal products or vitamin D_2_ from plant-based foods),^56^ dietary components such as fat content and dietary fiber,^56^^,^^57^ and modifications of VD in supplements and fortified foods.^58^ Moreover, endogenous elements, such as age,^59^ obesity,^60^ certain medical conditions related to gastrointestinal or renal complications,^61^ and genetic variations,^57^^,^^60^ are key determinants of an individuaĺs VD status. Notably, genetic variations, SNPs associated with VD status, are estimated to explain approximately 20% of the heritability of serum VD levels.^62^^,^^63^ It is worth mentioning that serum VD levels below 20–25 ng/mL indicate a state of deficiency.^63^ Vitamin D encompasses a multitude of functions, with its primary role revolving around bone mineralization.^64^ Recent years have seen a surge in studies investigating the beneficial impact of VD on various diseases, including cancer, cardiovascular diseases, diabetes mellitus, and obesity, among others.^65^ Together, these investigations underscore that VD engages in a broad spectrum of biological functions that extend beyond the realm of skeletal homeostasis.^66^ Indeed, the recognized actions of VD encompass processes such as cellular proliferation, inflammatory pathways, and modulation of the body's immune response.^64^ Due to its interplay with the immune system, where it modulates both innate and adaptive immune pathways, it is becoming increasingly evident that VD also influences infectious diseases initiated by viruses. For this reason, recent research has speculated on the significant role of this vitamin in severe acute respiratory diseases, particularly those triggered by the SARS-CoV-2 virus.^67^ In this context, numerous observational studies have explored the potential link between VD and COVID-19. The majority of these studies indicated a positive correlation between low serum levels of 25(OH)D and unfavorable health outcomes associated with COVID-19. Nevertheless, it is worth noting that these associations did not achieve statistical significance.^68^ To shed light on the causal relationship between VD and COVID-19, several randomized clinical trials have been carried out (**Table S3**^69–73^). The results of these studies have suggested that VD supplementation reduces symptom duration and hospitalization and decreases mechanical ventilation needs and readmissions. Although exogenous VD can play an important role in serum levels, contributing up to 10%–20% of total VD,^6^ especially in patients who are deficient in VD, this aspect was not considered in the studies reviewed. All MR studies used IVs constructed from SNPs associated with VD levels but did not account for determinants of VD levels or exogenous sources such as dietary intake and supplementation. In a recent systematic review of clinical trials on this topic, the study led by Meng et al^74^ confirmed that VD might have a beneficial effect on the severity of illnesses caused by SARS-CoV-2, particularly in individuals with VD deficiency. Interestingly, some studies have proposed that free VD, rather than VD levels in the blood reflecting both free VD and VD-binding protein (VDBP), could be connected to COVID-19 through immune pathways. Moreover, VDBP has been found to be depleted among patients with severe COVID-19, which increases the likelihood of low VD levels.^75^^,^^76^ This depletion of VDBP means that, even if the total levels of VD in the body are measured, they might not accurately reflect the amount of VD that is available to support the immune system. As a result, measured levels of VD deficiency might not reflect an individual’s immunological response to this disease.^77^ This suggests that free VD, rather than total VD, could be a more relevant biomarker when investigating the role of VD in immune function and COVID-19 disease outcomes. However, none of the MR studies included in this review took this into consideration since solely total VD levels were accounted for. Therefore, future research should focus on the levels of free VD and its interaction with VDBP to provide a clearer picture of how VD influences immune responses and potentially mitigates COVID-19 severity. Other micronutrients, however, have received little attention in this research domain (**Table S4**^78–84^). According to these studies, micronutrients such as vitamin C and zinc have no influence on COVID-19.

The aforementioned studies investigating the link between VD/micronutrients and COVID-19 and its outcomes were primarily experimental or observational, both susceptible to various biases. Causal inference studies via MR offer an advantage over observational studies by using genetic variants as IVs. This approach mimics a randomized controlled trial, mitigating biases such as confounding and reverse causation inherent to observational research studies. Over the past decade, advances in genetic technologies have enabled the identification of thousands of associations between genetic variation and relevant exposures, traits, and health outcomes. These genetic variations (SNPs, QTLs, etc) can be used as IVs to analyze the effect of modifiable exposures on diseases. Currently, MR is a widely used tool to search for causal associations between certain risk factors and many diseases, such as cancer,^85–87^ cardiovascular diseases,^88^^,^^89^ and COVID-19.^90^ In the present review, 12 studies on the association between VD/micronutrients and COVID-19, 4 studies on VD/micronutrients and inflammation-related markers, and 12 studies on inflammation and COVID-19 were identified.

COVID-19 and its severity have been related in several studies to inflammatory markers^91^^,^^92^ and micronutrients.^1^^,^^93^ However, as mentioned above, the specific role of these nutrients in disease development and their potential as adjuvant therapies remain unknown. Mendelian randomization studies on this issue are scarce but provide evidence on the impact that inflammatory markers and micronutrients have on the disease. In the present review, 12 studies on the association between VD/micronutrients and COVID-19, 4 studies on VD/micronutrients and inflammation-related markers, and 12 studies on inflammation and COVID-19 were identified. The results of this review show that VD and other micronutrients do not prevent COVID-19 or reduce disease outcomes. None of the studies addressed the possibility that free VD could play a role in the disease. Furthermore, none of these studies utilized linear or nonlinear MR to assess potential associations with COVID-19 at specific levels (ie, dose–response relationships). For instance, deficiency in the levels of these nutrients, particularly VD, could be linked to COVID-19 or related markers according to an earlier study conducted using biological databases and gene-pathway association analyses.^9^ In the present review, only the study by Zhou and Hyppönen^14^ evaluated the linearity of the association between VD and CRP to shed light on the potential dose-dependent effects between the 2. The results of this study showed that a VD deficiency increases the levels of CRP, suggesting that VD might exert varying effects depending on its levels. This finding might explain why no associations were observed between VD and other inflammatory markers, such as ACE2. It could also explain the absence of significant results in relation to COVID-19 outcomes. However, 2 of the studies that considered an IV of SNPs associated with VD deficiency reported a lack of association with COVID-19.^35^^,^^37^ It is important to highlight that the study by Patchen et al^37^ used SNPs from the GC, DHCR7, and CYP2R1 genes; however, these genes do not encompass all potential genes linked to VD deficiency. Likewise, the study by Amin et al^35^ considered 17 SNPs of a few genes to be associated with a deficiency of this nutrient. In fact, previous GWASs have identified over 20 SNPs associated with VD deficiency.^9^ It is essential to acknowledge that the failure to establish a robust IV can significantly impact the study's outcomes.^27^ In the case of the abovementioned studies,^35^^,^^37^ the IV might not have comprehensively represented the genetic basis of VD deficiency. It is also important to note that some studies took into account SNPs of VD transport and metabolism and of other pathways.^32^^,^^37^ However, no significant results were found in either of the 2 studies. Thus, the IV varied largely across the studies. Another aspect that could have influenced the observed results is the lack of consideration for ultraviolet B (UVB) radiation exposure, a crucial determinant of VD levels, in nearly all studies. Only 1 study that assessed the observational association between VD and COVID-19 considered UVB, although not in MR analyses.^33^ The SNPs of the VDR gene were also rarely considered to evaluate the association between VD and COVID-19.^5^ Finally, this review also shows that the outcome assessment relied in most studies on resources of the UKB and HGI studies, since these studies were first in accounting for genome data of patients with COVID-19. Thus, while MR studies and meta-analyses combining their results do not support an association between VD and COVID-19 outcomes, uncertainties persist due to methodological variations and the intricate nature of the relationship between VD levels and this disease.

The methodological quality of the studies, assessed by the MR-STROBE guidelines, was rated high in 26 studies and low in the remaining studies. Studies with high quality were mostly those accounting for the association between VD and COVID-19.^32^^,^^34^^,^^38^^,^^40–42^ However, among the MR studies of this review, few evaluated reverse causality between micronutrients/VD and inflammation markers or COVID-19 through bidirectional MR (ie, the exposure–outcome associations from both sides).^14^^,^^44^^,^^53^ Reverse causality occurs when an exposure is modified by the outcome, unlike what is expected (eg, a disease alters a risk factor studied for this disease).^94^ Hence, determining the causative direction is crucial to discern whether deficient levels of VD or elevated inflammation markers contribute to COVID-19, or vice versa. According to the review, the direction of these associations remains unknown.

Several strengths characterize this review. First, it shows a comprehensive appraisal of prior MR studies on micronutrients/VD and inflammatory markers and their associations with specific outcomes of COVID-19. Clear and predefined evaluation criteria were applied to scrutinize the studies on this topic. Second, the review presents results on large-scale study populations with genetic data from the UKB, HGI, and other cohorts. Results of studies from diverse ethnic groups, encompassing European,^32^^,^^45^^,^^46^^,^^49–52^^,^^54^^,^^95^ Asian,^45^ African,^45^ and Latinos,^50^^,^^52^ were included, although genetic ancestry data were lacking in certain studies.^47^^,^^48^^,^^53^^,^^55^ Therefore, the results are not entirely generalizable. Moreover, given that VD deficiency is more prevalent among Black populations,^96^ more studies are needed to confirm the absence of a relationship between this nutrient and COVID-19 disease. Vitamin D deficiency can differentially impact various diseases depending on race and ethnic origin. Deficient VD levels may contribute to significant variations in the prevalence and severity of conditions such as osteoporosis, cardiovascular diseases, type 2 diabetes, autoimmune diseases, respiratory infections, and certain types of cancer. Differences in VD levels and the prevalence of VD deficiency according to race may explain these variations.^97^^,^^98^ It is crucial to consider these differences in research, including studies on COVID-19, as it is a respiratory illness with a potent inflammatory component that also may vary greatly depending on the race and ethnic background of the affected individuals. Indeed, previous studies, such as the one conducted by Gibbons et al,^99^ have analyzed individuals from different racial backgrounds. These studies concluded that Black individuals experienced a greater reduction in COVID-19 infection rates associated with VD supplementation compared with White individuals, relative to controls. Third, to existing knowledge, no systematic review with meta-analysis has previously explored the causal relationship between micronutrients and COVID-19, overcoming common biases inherent to observational studies. Thus, this review sheds light on this subject and provides, for the first time, valuable insights into this relationship. Last, to ensure the inclusivity of relevant MR studies, the search strategy adhered to the PRISMA guidelines.^25^

However, this review also has some limitations to note. It was not possible to analyze in depth the association between certain micronutrients (minerals and vitamins other than VD) and COVID-19 outcomes given the limited number of causal inference studies on these nutrients. Additionally, variability in study designs and IV assessment might have impacted the consistency of the findings. Furthermore, there might be some overlap between data from the UKB and other study populations among the studies included in this review. Therefore, care was taken to combine the results of studies that relied on different study populations for IV assessment at the exposure level (eg, IV-VD assessment in different studies). It is worth noting that the presence of pleiotropy cannot be entirely dismissed since not all studies evaluated its effect when estimating causal effects. The variability in the methodological quality among the studies could have influenced the accuracy of the conclusions drawn in this review. Therefore, a rigorous assessment of the methodological quality of each study was conducted to ensure the reliability of the reported results. Finally, as in any other systematic review, the possibility of publication bias might have affected the overall assessment of the causal relationships.

## CONCLUSION

Causal inference studies based on MR approaches on the association between micronutrients/VD and COVID-19 outcomes or related inflammatory markers showed methodological variations; nevertheless, these studies do not support a causal link between these factors. However, according to these studies, VD deficiency correlates with an increased inflammatory state, potentially impacting COVID-19 disease risk and associated outcomes. Additionally, various inflammatory markers and circulating proteins, including ACE2, are causally connected to COVID-19 disease risk. Thus, whether inflammation mediates the relationship between VD and COVID-19 remains to be elucidated in future MR studies exploring micronutrient associations with COVID-19 outcomes.

## Supplementary Material

nuae152_Supplementary_Data

## References

[1] Bassatne A , Basbous M, Chakhtoura M, El Zein O, Rahme M, El-Hajj Fuleihan G. The link between COVID-19 and VItamin D (VIVID): a systematic review and meta-analysis. Metabolism. 2021;119:154753. 10.1016/j.metabol.2021.154753 33774074 PMC7989070

[2] Chen N , Zhou M, Dong X, et al Epidemiological and clinical characteristics of 99 cases of 2019 novel coronavirus pneumonia in Wuhan, China: a descriptive study. Lancet. 2020;395:507-513. 10.1016/S0140-6736(20)30211-7 32007143 PMC7135076

[3] Huang C , Wang Y, Li X, et al Clinical features of patients infected with 2019 novel coronavirus in Wuhan, China. Lancet. 2020;395:497-506. 10.1016/S0140-6736(20)30183-5 31986264 PMC7159299

[4] Gombart AF , Pierre A, Maggini S. A review of micronutrients and the immune system—working in harmony to reduce the risk of infection. Nutrients. 2020;12:236. 10.3390/nu12010236 31963293 PMC7019735

[5] Kotur N , Stankovic B, Pavlovic S. Micronutrients, genetics and COVID-19. Curr Opin Clin Nutr Metab Care. 2023;26:309-315. 10.1097/MCO.0000000000000942 37144461

[6] Holick MF. Vitamin D and bone health: what vitamin D can and cannot do. Adv Food Nutr Res. 2024;109:43-66. 10.1016/bs.afnr.2024.04.002 38777417

[7] Cardwell G , Bornman J, James A, Black L. A review of mushrooms as a potential source of dietary vitamin D. Nutrients. 2018;10:1498. 10.3390/nu10101498 30322118 PMC6213178

[8] Chiodini I , Gatti D, Soranna D, et al Vitamin D status and SARS-CoV-2 infection and COVID-19 clinical outcomes. Front Public Health. 2021;9:736665. 10.3389/fpubh.2021.736665 35004568 PMC8727532

[9] Alcalá-Santiago Á , Rodríguez-Barranco M, Rava M, et al Vitamin D deficiency and COVID-19: a biological database study on pathways and gene-disease associations. Int J Mol Sci. 2022;23:14256. 10.3390/ijms232214256 36430729 PMC9699081

[10] Annweiler G , Corvaisier M, Gautier J, et al Vitamin D supplementation associated to better survival in hospitalized frail elderly COVID-19 patients: the GERIA-COVID quasi-experimental study. Nutrients. 2020;12:3377. 10.3390/nu12113377 33147894 PMC7693938

[11] Entrenas Castillo M , Entrenas Costa LM, Vaquero Barrios JM, et al Effect of calcifediol treatment and best available therapy versus best available therapy on intensive care unit admission and mortality among patients hospitalized for COVID-19: a pilot randomized clinical study. J Steroid Biochem Mol Biol. 2020;203:105751. 10.1016/j.jsbmb.2020.105751 32871238 PMC7456194

[12] Munshi R , Hussein MH, Toraih EA, et al Vitamin D insufficiency as a potential culprit in critical COVID‐19 patients. J Med Virol. 2021;93:733-740. 10.1002/jmv.26360 32716073

[13] Hu B , Huang S, Yin L. The cytokine storm and COVID‐19. J Med Virol. 2021;93:250-256. 10.1002/jmv.26232 32592501 PMC7361342

[14] Zhou A , Hyppönen E. Vitamin D deficiency and C-reactive protein: a bidirectional Mendelian randomization study. Int J Epidemiol. 2023;52:260-271. 10.1093/ije/dyac087 35579027 PMC9908047

[15] Gusev E , Sarapultsev A, Solomatina L, Chereshnev V. SARS-CoV-2-specific immune response and the pathogenesis of COVID-19. Int J Mol Sci. 2022;23:1716. 10.3390/ijms23031716 35163638 PMC8835786

[16] Shenoy S. Gut microbiome, vitamin D, ACE2 interactions are critical factors in immune-senescence and inflammaging: key for vaccine response and severity of COVID-19 infection. Inflamm Res. 2022;71:13-26. 10.1007/s00011-021-01510-w 34738147 PMC8568567

[17] Moreno-Montañés J , Gándara E, Moreno-Galarraga L, et al ACE-vitamin index and risk of glaucoma: the SUN project. Nutrients. 2022;14:5129. 10.3390/nu14235129 36501162 PMC9735492

[18] Burgess S , Scott RA, Timpson NJ, Davey Smith G, Thompson SG; EPIC- InterAct Consortium. Using published data in Mendelian randomization: a blueprint for efficient identification of causal risk factors. Eur J Epidemiol. 2015;30:543-552. 10.1007/s10654-015-0011-z 25773750 PMC4516908

[19] Lawler T , Warren Andersen S. Serum 25-hydroxyvitamin D and cancer risk: a systematic review of Mendelian randomization studies. Nutrients. 2023;15:422. 10.3390/nu15020422 36678292 PMC9865859

[20] Ran S , Qiu X, Liu B. Causal relationship between asthma-related diseases and the risk of COVID-19: A 2-sample Mendelian randomization study. J Allergy Clin Immunol. 2023;152:814-815. 10.1016/j.jaci.2023.04.024 37436347

[21] Chen H , Ye B, Su W, et al The causal role of gut microbiota in susceptibility and severity of COVID‐19: a bidirectional Mendelian randomization study. J Med Virol. 2023;95:e28880. 10.1002/jmv.28880 37409643

[22] Manousaki D , Harroud A, Mitchell RE, et al Vitamin D levels and risk of type 1 diabetes: a Mendelian randomization study. PLoS Med. 2021;18:e1003536. 10.1371/journal.pmed.1003536 33630834 PMC7906317

[23] Flatby HM , Ravi A, Damås JK, Solligård E, Rogne T. Circulating levels of micronutrients and risk of infections: a Mendelian randomization study. BMC Med. 2023;21:84. 10.1186/s12916-023-02780-3 36882828 PMC9993583

[24] Papadimitriou N , Dimou N, Gill D, et al Genetically predicted circulating concentrations of micronutrients and risk of breast cancer: a Mendelian randomization study. Int J Cancer. 2021;148:646-653. 10.1002/ijc.33246 32761610 PMC8268064

[25] Moher D , Liberati A, Tetzlaff J, Altman DG; PRISMA Group. Preferred Reporting Items for Systematic Reviews and Meta-Analyses: the PRISMA statement. BMJ. 2009;339:b2535. 10.1136/bmj.b2535 19622551 PMC2714657

[26] Page MJ , McKenzie JE, Bossuyt PM, et al The PRISMA 2020 statement: an updated guideline for reporting systematic reviews. BMJ. 2021;372:n71. 10.1136/bmj.n71 33782057 PMC8005924

[27] Burgess S , Davey Smith G, Davies NM, et al Guidelines for performing Mendelian randomization investigations: update for summer 2023. Wellcome Open Res. 2019;4:186. 10.12688/wellcomeopenres.15555.3 32760811 PMC7384151

[28] Davey Smith G , Davies NM, Dimou N, et al STROBE-MR: guidelines for strengthening the reporting of Mendelian randomization studies. PeerJ Prepr. 2019;7:e27857v1.

[29] R Core Team. R: a language and environment for statistical computing. R Foundation for Statistical Computing; 2022. Accessed July 25, 2023. https://www.R-project.org

[30] Viechtbauer W. Conducting meta-analyses in R with the metafor package. J Stat Soft. 2010;36: Doi:10.18637/jss.v036.i03

[31] Au Yeung SL , Wong THT, He B, Luo S, Kwok KO. Does ACE2 mediate the detrimental effect of exposures related to COVID‐19 risk: a Mendelian randomization investigation. J Med Virol. 2023;95:e28205. 10.1002/jmv.28205 36217700 PMC9874514

[32] Butler-Laporte G , Nakanishi T, Mooser V, et al Vitamin D and COVID-19 susceptibility and severity in the COVID-19 host genetics initiative: a Mendelian randomization study. PLoS Med. 2021;18:e1003605. 10.1371/journal.pmed.1003605 34061844 PMC8168855

[33] Li X , van Geffen J, van Weele M, et al An observational and Mendelian randomisation study on vitamin D and COVID-19 risk in UK Biobank. Sci Rep. 2021;11:18262. 10.1038/s41598-021-97679-5 34521884 PMC8440633

[34] Cui Z , Tian Y. Using genetic variants to evaluate the causal effect of serum vitamin D concentration on COVID-19 susceptibility, severity and hospitalization traits: a Mendelian randomization study. J Transl Med. 2021;19:300. 10.1186/s12967-021-02973-5 34246301 PMC8271325

[35] Amin HA , Drenos F. No evidence that vitamin D is able to prevent or affect the severity of COVID-19 in individuals with European ancestry: a Mendelian randomisation study of open data. BMJ Nutr Prev Health. 2021;4:42-48. 10.1136/bmjnph-2020-000151 PMC7798425 34308111

[36] Liu D , Tian QY, Zhang J, et al Association between 25 hydroxyvitamin D concentrations and the risk of COVID-19: a Mendelian randomization study. Biomed Environ Sci. 2021;34:750-754. 10.3967/bes2021.104 34530967 PMC8485511

[37] Patchen BK , Clark AG, Gaddis N, Hancock DB, Cassano PA. Genetically predicted serum vitamin D and COVID-19: a Mendelian randomisation study. BMJ Nutr Prev Health. 2021;4:213-225. 10.1136/bmjnph-2021-000255 PMC8098235 34308129

[38] Qiu S , Zheng K, Hu Y, Liu G. Genetic correlation, causal relationship, and shared loci between vitamin D and COVID‐19: a genome‐wide cross‐trait analysis. J Med Virol. 2023;95:e28780. 10.1002/jmv.28780 37212302

[39] Hui LL , Nelson EAS, Lin SL, Zhao JV. The role of vitamin C in pneumonia and COVID-19 infection in adults with European ancestry: a Mendelian randomisation study. Eur J Clin Nutr. 2022;76:588-591. 10.1038/s41430-021-00993-4 34462559 PMC8404179

[40] Mohus RM , Flatby H, Liyanarachi KV, et al Iron status and the risk of sepsis and severe COVID-19: a two-sample Mendelian randomization study. Sci Rep. 2022;12:16157. 10.1038/s41598-022-20679-6 36171422 PMC9516524

[41] Sobczyk MK , Gaunt TR. The effect of circulating zinc, selenium, copper and vitamin K1 on COVID-19 outcomes: a Mendelian randomization study. Nutrients. 2022;14:233. 10.3390/nu14020233 35057415 PMC8780111

[42] Daniel N , Bouras E, Tsilidis KK, Hughes DJ. Genetically predicted circulating concentrations of micronutrients and COVID-19 susceptibility and severity: a Mendelian randomization study. Front Nutr. 2022;9:842315. 10.3389/fnut.2022.842315 35558754 PMC9085481

[43] Palaniswamy S , Gill D, De Silva NM, et al Could vitamin D reduce obesity-associated inflammation? Observational and Mendelian randomization study. Am J Clin Nutr. 2020;111:1036-1047. 10.1093/ajcn/nqaa056 32232398 PMC7198294

[44] Liefaard MC , Ligthart S, Vitezova A, et al Vitamin D and C-reactive protein: a Mendelian randomization study. PLoS One. 2015;10: E 0131740. 10.1371/journal.pone.0131740 PMC4492676 26147588

[45] Kousathanas A , Pairo-Castineira E, Rawlik K, et al COVID-19 Human Genetics Initiative. Whole-genome sequencing reveals host factors underlying critical COVID-19. Nature. 2022;607:97-103. 10.1038/s41586-022-04576-6 35255492 PMC9259496

[46] Richardson TG , Fang S, Mitchell RE, Holmes MV, Davey Smith G. Evaluating the effects of cardiometabolic exposures on circulating proteins which may contribute to severe SARS-CoV-2. EBioMedicine. 2021;64:103228. 10.1016/j.ebiom.2021.103228 33548839 PMC7857697

[47] Gaziano L , Giambartolomei C, Pereira AC, et al VA Million Veteran Program COVID-19 Science Initiative. Actionable druggable genome-wide Mendelian randomization identifies repurposing opportunities for COVID-19. Nat Med. 2021;27:668-676. 10.1038/s41591-021-01310-z 33837377 PMC7612986

[48] Zheng M. ACE2 and COVID-19 susceptibility and severity. Aging Dis. 2022;13:360-372. 10.14336/AD.2021.0805 35371596 PMC8947832

[49] Yang Z , Macdonald-Dunlop E, Chen J, et al IMI-DIRECT Consortium. Genetic landscape of the ACE2 coronavirus receptor. Circulation. 2022;145:1398-1411. 10.1161/CIRCULATIONAHA.121.057888 35387486 PMC9047645

[50] Butler-Laporte G , Nakanishi T, Mooser V, et al The effect of angiotensin-converting enzyme levels on COVID-19 susceptibility and severity: a Mendelian randomization study. Int J Epidemiol. 2021;50:75-86. 10.1093/ije/dyaa229 33349849 PMC7799043

[51] Gill D , Arvanitis M, Carter P, et al ACE inhibition and cardiometabolic risk factors, lung ACE2 and TMPRSS2 gene expression, and plasma ACE2 levels: a Mendelian randomization study. R Soc Open Sci. 2020;7:200958. 10.1098/rsos.200958 33391794 PMC7735342

[52] Sood T , Perrot N, Chong M, et al Biomarkers associated with severe COVID-19 among populations with high cardiometabolic risk. JAMA Netw Open. 2023;6: E 2325914. 10.1001/jamanetworkopen.2023.25914 PMC10375306 37498601

[53] Li M , Yeung CHC, Schooling CM. Circulating cytokines and coronavirus disease: a bi-directional Mendelian randomization study. Front Genet. 2021;12:680646. 10.3389/fgene.2021.680646 34163532 PMC8215612

[54] Wang K , Qu M, Ding L, et al Liver and kidney function biomarkers, blood cell traits and risk of severe COVID-19: a Mendelian randomization study. Front Genet. 2021;12:647303. 10.3389/fgene.2021.647303 34122505 PMC8191502

[55] Zhu H , Zheng F, Li L, et al A Chinese host genetic study discovered IFNs and causality of laboratory traits on COVID-19 severity. iScience. 2021;24:103186. 10.1016/j.isci.2021.103186 34608450 PMC8481128

[56] Weber F. Absorption mechanisms for fat-soluble vitamins and the effect of other food constituents. Prog Clin Biol Res. 1981;77:119-135. 7038701

[57] Niramitmahapanya S , Harris SS, Dawson-Hughes B. Type of dietary fat is associated with the 25-hydroxyvitamin D3 increment in response to vitamin D supplementation. J Clin Endocrinol Metab. 2011;96:3170-3174. 10.1210/jc.2011-1518 21816779 PMC3200243

[58] Natri AM , Salo P, Vikstedt T, et al Bread fortified with cholecalciferol increases the serum 25-hydroxyvitaminD concentration in women as effectively as a cholecalciferol supplement. J Nutr. 2006;136:123-127. 10.1093/jn/136.1.123 16365070

[59] Clemens TL , Zhou XY, Myles M, Endres D, Lindsay R. Serum vitamin D_2_ and vitamin D_3_ metabolite concentrations and absorption of vitamin D_2_ in elderly subjects. J Clin Endocrinol Metab. 1986;63:656-660. 10.1210/jcem-63-3-656 3488327

[60] Wortsman J , Matsuoka LY, Chen TC, Lu Z, Holick MF. Decreased bioavailability of vitamin D in obesity. Am J Clin Nutr. 2000;72:690-693. 10.1093/ajcn/72.3.690 10966885

[61] Farraye FA , Nimitphong H, Stucchi A, et al Use of a novel vitamin D bioavailability test demonstrates that vitamin D absorption is decreased in patients with quiescent Crohnʼs disease. Inflamm Bowel Dis. 2011;17:2116-2121. 10.1002/ibd.21595 21910173

[62] Manousaki D , Mitchell R, Dudding T, et al Genome-wide association study for vitamin D levels reveals 69 independent loci. Am J Hum Genet. 2020;106:327-337. 10.1016/j.ajhg.2020.01.017 32059762 PMC7058824

[63] Holick MF , Chen TC. Vitamin D deficiency: a worldwide problem with health consequences. Am J Clin Nutr. 2008;87:1080S-1086S. 10.1093/ajcn/87.4.1080S 18400738

[64] Pike JW , Christakos S. Biology and mechanisms of action of the vitamin D hormone. Endocrinol Metab Clin North Am. 2017;46:815-843. 10.1016/j.ecl.2017.07.001 29080638 PMC5762112

[65] Furuie IN , Mauro MJJ, Petruzziello S, et al Two threshold levels of vitamin D and the prevalence of comorbidities in outpatients of a tertiary hospital. Osteoporos Int. 2018;29:433-440. 10.1007/s00198-017-4299-2 29143130

[66] Gil Á , Plaza-Diaz J, Mesa MD. Vitamin D: classic and novel actions. Ann Nutr Metab. 2018;72:87-95. 10.1159/000486536 29346788

[67] Ghasemian R , Shamshirian A, Heydari K, et al The role of vitamin D in the age of COVID‐19: a systematic review and meta‐analysis. Int J Clin Pract. 2021;75:e14675. 10.1111/ijcp.14675 34322971 PMC8420549

[68] Bignardi PR , Castello P de A, Aquino B de M, Delfino VDA. Is the vitamin D status of patients with COVID-19 associated with reduced mortality? A systematic review and meta-analysis. Arch Endocrinol Metab. 2023;67:276-288. 10.20945/2359-3997000000588 36913680 PMC10689034

[69] Alcala-Diaz JF , Limia-Perez L, Gomez-Huelgas R, et al Calcifediol treatment and hospital mortality due to COVID-19: a cohort study. Nutrients. 2021;13:1760. 10.3390/nu13061760 34064175 PMC8224356

[70] Murai IH , Fernandes AL, Sales LP, et al Effect of a single high dose of vitamin D_3_ on hospital length of stay in patients with moderate to severe COVID-19. JAMA. 2021;325:1053-1060. 10.1001/jama.2020.26848 33595634 PMC7890452

[71] Sabico S , Enani MA, Sheshah E, et al Effects of a 2-week 5000 IU versus 1000 IU vitamin D3 supplementation on recovery of symptoms in patients with mild to moderate Covid-19: a randomized clinical trial. Nutrients. 2021;13:2170. 10.3390/nu13072170 34202578 PMC8308273

[72] Annweiler C , Beaudenon M, Simon R, et al GERIA-COVID Study Group. Vitamin D supplementation prior to or during COVID-19 associated with better 3-month survival in geriatric patients: extension phase of the GERIA-COVID study. J Steroid Biochem Mol Biol. 2021;213:105958. 10.1016/j.jsbmb.2021.105958 34332023 PMC8319044

[73] Elamir YM , Amir H, Lim S, et al A randomized pilot study using calcitriol in hospitalized COVID-19 patients. Bone. 2022;154:116175. 10.1016/j.bone.2021.116175 34508882 PMC8425676

[74] Meng J , Li X, Liu W, et al The role of vitamin D in the prevention and treatment of SARS-CoV-2 infection: a meta-analysis of randomized controlled trials. Clin Nutr. 2023;42:2198-2206. 10.1016/j.clnu.2023.09.008 37802017

[75] Subramanian S , Rhodes JM, Taylor JM, et al Vitamin D, vitamin D–binding protein, free vitamin D and COVID-19 mortality in hospitalized patients. Am J Clin Nutr. 2022;115:1367-1377. 10.1093/ajcn/nqac027 35102371 PMC8903333

[76] Faniyi AA , Lugg ST, Faustini SE, et al Genetic polymorphisms, vitamin D binding protein and vitamin D deficiency in COVID-19. Eur Respir J. 2021;57:2100653. 10.1183/13993003.00653-2021 33888522 PMC8100329

[77] Saponaro F , Franzini M, Okoye C, et al Is there a crucial link between vitamin D status and inflammatory response in patients with COVID-19? Front Immunol. 2021;12:745713. 10.3389/fimmu.2021.745713 35140702 PMC8818986

[78] Thomas S , Patel D, Bittel B, et al Effect of high-dose zinc and ascorbic acid supplementation vs usual care on symptom length and reduction among ambulatory patients with SARS-CoV-2 infection. JAMA Netw Open. 2021;4: E 210369. 10.1001/jamanetworkopen.2021.0369 PMC7881357 33576820

[79] Xing Y , Zhao B, Yin L, et al Vitamin C supplementation is necessary for patients with coronavirus disease: an ultra-high-performance liquid chromatography-tandem mass spectrometry finding. J Pharm Biomed Anal. 2021;196:113927. 10.1016/j.jpba.2021.113927 33549875 PMC7839397

[80] Jamali Moghadam Siahkali S , Zarezade B, Koolaji S, et al Safety and effectiveness of high-dose vitamin C in patients with COVID-19: a randomized open-label clinical trial. Eur J Med Res. 2021;26:20. 10.1186/s40001-021-00490-1 33573699 PMC7877333

[81] Kumari P , Dembra S, Dembra P, et al The role of vitamin C as adjuvant therapy in COVID-19. Cureus. 2020;12:e11779. 10.7759/cureus.11779. 33409026 PMC7779177

[82] Majidi N , Rabbani F, Gholami S, et al The effect of vitamin C on pathological parameters and survival duration of critically ill coronavirus disease 2019 patients: a randomized clinical trial. Front Immunol. 2021;12:717816. 10.3389/fimmu.2021.717816 34975830 PMC8714637

[83] Patel O , Chinni V, El‐Khoury J, et al A pilot double‐blind safety and feasibility randomized controlled trial of high‐dose intravenous zinc in hospitalized COVID‐19 patients. J Med Virol. 2021;93:3261-3267. 10.1002/jmv.26895 33629384 PMC8014767

[84] Abd-Elsalam S , Soliman S, Esmail ES, et al Do zinc supplements enhance the clinical efficacy of hydroxychloroquine? A randomized, multicenter trial. Biol Trace Elem Res. 2021;199:3642-3646. 10.1007/s12011-020-02512-1 33247380 PMC7695238

[85] Zhou W , Liu G, Hung RJ, et al Causal relationships between body mass index, smoking and lung cancer: univariable and multivariable Mendelian randomization. Int J Cancer. 2021;148:1077-1086. 10.1002/ijc.33292 32914876 PMC7845289

[86] Chan II , Kwok MK, Schooling CM. Blood pressure and risk of cancer: a Mendelian randomization study. BMC Cancer. 2021;21:1338. 10.1186/s12885-021-09067-x 34915881 PMC8675492

[87] Molina-Montes E , Coscia C, Gómez-Rubio P, et al PanGenEU Study Investigators. Deciphering the complex interplay between pancreatic cancer, diabetes mellitus subtypes and obesity/BMI through causal inference and mediation analyses. Gut. 2021;70:319-329. https://doi.org/ 10.1136/gutjnl-2019-319990. 32409590 10.1136/gutjnl-2019-319990

[88] Larsson SC , Burgess S, Mason AM, Michaëlsson K. Alcohol consumption and cardiovascular disease. Circ Genom Precis Med. 2020;13:e002814. Doi:10.1161/CIRCGEN.119.002814 32367730 PMC7299220

[89] Chen D , Zhang Y, Yidilisi A, Xu Y, Dong Q, Jiang J. Causal associations between circulating adipokines and cardiovascular disease: a Mendelian randomization study. J Clin Endocrinol Metab. 2022;107:e2572-e2580. 10.1210/clinem/dgac048 35134201 PMC9113792

[90] Luo S , Liang Y, Wong THT, Schooling CM, Au Yeung SL. Identifying factors contributing to increased susceptibility to COVID-19 risk: a systematic review of Mendelian randomization studies. Int J Epidemiol. 2022;51:1088-1105. 10.1093/ije/dyac076 35445260 PMC9047195

[91] Hariyanto TI , Japar KV, Kwenandar F, et al Inflammatory and hematologic markers as predictors of severe outcomes in COVID-19 infection: a systematic review and meta-analysis. Am J Emerg Med. 2021;41:110-119. 10.1016/j.ajem.2020.12.076 33418211 PMC7831442

[92] Lai YJ , Liu SH, Manachevakul S, Lee TA, Kuo CT, Bello D. Biomarkers in long COVID-19: a systematic review. Front Med (Lausanne). 2023;10:1085988. 10.3389/fmed.2023.1085988 36744129 PMC9895110

[93] Migliorini F , Vaishya R, Eschweiler J, Oliva F, Hildebrand F, Maffulli N. Vitamins C and D and COVID-19 susceptibility, severity and progression: an evidence based systematic review. Medicina (Kaunas). 2022;58:941. 10.3390/medicina58070941 35888660 PMC9318801

[94] Sekula P , Del Greco M F, Pattaro C, Köttgen A. Mendelian randomization as an approach to assess causality using observational data. J Am Soc Nephrol. 2016;27:3253-3265. 10.1681/ASN.2016010098 27486138 PMC5084898

[95] Liu H , Xin J, Cai S, Jiang X. Mendelian randomization analysis provides causality of smoking on the expression of ACE2, a putative SARS-CoV-2 receptor. Elife. 2021;10: Doi:10.7554/eLife.64188 PMC8282334 34227468

[96] Meltzer DO , Best TJ, Zhang H, Vokes T, Arora VM, Solway J. Association of vitamin D levels, race/ethnicity, and clinical characteristics with COVID-19 test results. JAMA Netw Open. 2021;4:e214117. 10.1001/jamanetworkopen.2021.4117 33739433 PMC7980095

[97] Johansson E , Biagini JM, Martin LJ, et al Vitamin D, skin filaggrin, allergic sensitization, and race. Ann Allergy Asthma Immunol. 2022;128:399-407.e3. 10.1016/j.anai.2022.01.017 35081436 PMC9109635

[98] Sawicki CM , Van Rompay MI, Au LE, Gordon CM, Sacheck JM. Sun-exposed skin color is associated with changes in serum 25-hydroxyvitamin D in racially/ethnically diverse children. J Nutr. 2016;146:751-757. 10.3945/jn.115.222505 26936138 PMC4807642

[99] Gibbons JB , Norton EC, McCullough JS, et al Association between vitamin D supplementation and COVID-19 infection and mortality. Sci Rep. 2022;12:19397. 10.1038/s41598-022-24053-4 36371591 PMC9653496

