# Supplementary material for: Micronutrients, Vitamin D, and Inflammatory Biomarkers in COVID-19: A Systematic Review and Meta-analysis of Causal Inference Studies
Source: Nutr Rev. 2024 Oct 24;83(7):e1383–405. doi: 10.1093/nutrit/nuae152 (PMC12166185; doi:10.1093/nutrit/nuae152)
Supplement: nuae152_Supplementary_Data [file nuae152_supplementary_data.zip › nuae152_Supplementary_Data/Alcala-Santiago et al._Suppl mat. 4.docx]

**Index**

1. Search strategy
2. Table S1. MR strobe checklist for items related to the methods according to MR-STROBE guidelines.
3. Table S2. MR strobe checklist for items related to reporting of the results and discussion according to MR-STROBE guidelines.
4. Table S3. Randomized controlled studies on the impact of vitamin D on COVID-19 disease.
5. Table S4. Randomized controlled studies on the impact of micronutrients other than vitamin D on COVID-19 disease.
6. **Search strategy**

Search 1: (((micronutrients [MeSH Terms]) OR (micronutrients)) OR ((vitamin d) OR (vitamin d[MeSH Terms])) OR ((Cholecalciferol) OR (cholecalciferol[MeSH Terms]))) AND (((COVID-19) OR (COVID-19[MeSH Terms])) OR ((SARS-CoV-2) OR (SARS-CoV-2[MeSH Terms])) OR ((2019-nCoV) OR (2019-nCoV[MeSH Terms])) OR ((nCoV-19) OR (nCoV-19[MeSH Terms]))) AND ((Mendelian Randomization) OR (Mendelian Randomization[MeSH Terms]))

Search 2: ((micronutrients [MeSH Terms]) OR (micronutrients) OR (vitamin d) OR (vitamin d[MeSH Terms]) OR (Cholecalciferol) OR (cholecalciferol[MeSH Terms])) AND ((inflammatory markers) OR (inflammatory markers[MeSH Terms]) OR (in-flammatory biomarkers) OR (inflammatory biomarkers[MeSH Terms])) AND ((Men-delian Randomization) OR (Mendelian Randomization[MeSH Terms]))

Search 3: ((inflammatory markers) OR (inflammatory markers [MeSH Terms]) OR (inflammatory biomarkers) OR (inflammatory biomarkers [MeSH Terms])) AND ((COVID-19) OR (COVID-19[MeSH Terms]) OR (SARS-CoV-2) OR (SARS-CoV-2[MeSH Terms]) OR (2019-nCoV) OR (2019-nCoV [MeSH Terms]) OR (nCoV-19) OR (nCoV-19[MeSH Terms])) AND ((Mendelian Randomization) OR (Mendelian Randomization [MeSH Terms]))

Additional searches were added to grasp studies covering the ACE2 system in relation to COVID-19 and micronutrients, including VD.

Search 4: ((micronutrients [MeSH Terms]) OR (micronutrients) OR (vitamin d) OR (vitamin d [MeSH Terms]) OR (cholecalciferol) OR (cholecalciferol [MeSH Terms])) AND ((ACE2) OR (ACE2 [MeSH Terms]) OR (angiotensin-converting enzyme 2) OR (angiotensin-converting enzyme 2 [MeSH Terms])) AND ((Mendelian randomization) OR (Mendelian randomization [MeSH Terms]))

Search 5: ((COVID-19) OR (COVID-19[MeSH Terms]) OR (SARS-CoV-2) OR (SARS-CoV-2[MeSH Terms]) OR (2019-nCoV) OR (2019-nCoV [MeSH Terms]) OR (nCoV-19) OR (nCoV-19[MeSH Terms])) AND ((ACE2) OR (ACE2[MeSH Terms]) OR (Angiotensin-Converting Enzyme 2) OR (Angiotensin-Converting Enzyme 2[MeSH Terms])) AND ((Mendelian Randomization) OR (Mendelian Randomization [MeSH Terms]

1. **Table S1.** MR strobe checklist for items related to the methods according to MR-STROBE guidelines.

| items | 1 | 2 | 3 | 4 | 5 | 6 | 7 | 8 | 9 | 10 | 11 | 12 | 13 | 14 |
| --- | --- | --- | --- | --- | --- | --- | --- | --- | --- | --- | --- | --- | --- | --- |
| Study | Setting | Participants | Measurement | Assessment methods/criteria | Ethics | IV assumptions | Quantitative variables | Genetic variants | MR estimator | Missing data | Multiple testing | Assumptions verification | Sensitivity analyses | Software |
| Au Yeung et al. (2022)^31^ | N | N | Y | Y | NA | Y | Y | Y | Y | N | NA | Y | Y | Y |
| Butler-Laporte et al. (2021)^32^ | Y | Y | Y | Y | Y | Y | Y | Y | Y | Y | NA | N | Y | Y |
| Li et al. (2021)^33^ | Y | Y | Y | Y | NA | N | Y | Y | Y | Y | Y | NA | Y | Y |
| Cui et al. (2021)^34^ | Y | Y | Y | Y | NA | Y | Y | Y | Y | Y | Y | Y | Y | N |
| Amin et al. (2021)^35^ | Y | Y | Y | Y | NA | N | Y | Y | Y | Y | NA | N | U | Y |
| Liu D et al. (2021)^36^ | Y | Y | Y | Y | NA | U | Y | Y | Y | N | NA | U | Y | Y |
| Patchen et al. (2021)^37^ | Y | Y | Y | Y | NA | Y | Y | N | Y | N | Y | Y | Y | Y |
| Qiu et al. (2023)^38^ | Y | Y | Y | Y | NA | Y | Y | Y | Y | Y | Y | N | Y | Y |
| Hui et al. (2021)^39^ | Y | Y | Y | Y | NA | N | Y | Y | Y | N | NA | N | Y | Y |
| Mohus et al. (2022)^40^ | Y | Y | Y | Y | Y | Y | Y | Y | Y | Y | NA | Y | Y | Y |
| Sobczyk et al. (2022)^41^ | N | N | Y | Y | NA | Y | Y | Y | Y | Y | Y | Y | Y | N |
| Daniel et al. (2022)^42^ | Y | Y | Y | Y | NA | Y | Y | Y | Y | Y | Y | Y | Y | Y |
| Zhou et al. (2022)^14^ | Y | Y | Y | Y | Y | N | Y | Y | Y | Y | Y | N | Y | Y |
| Palaniswamy et al.(2020)^43^ | Y | Y | Y | Y | Y | N | Y | Y | Y | N | Y | N | Y | Y |
| Liefaard et al. (2015)^44^ | Y | Y | Y | Y | NA | N | Y | Y | Y | Y | Y | N | Y | Y |
| Kousathanas et al.(2022)^45^ | Y | N | Y | Y | Y | U | Y | N | Y | Y | Y | N | Y | Y |
| Richardson et al.(2021)^46^ | Y | N | Y | Y | NA | Y | Y | N | Y | N | Y | Y | Y | Y |
| Gaziano et al. (2021)^47^ | Y | Y | Y | Y | NA | U | Y | N | Y | N | Y | N | N | N |
| Zheng et al.(2022)^48^ | Y | Y | Y | Y | NA | N | Y | Y | Y | N | N | N | N | Y |
| Yang et al. (2022)^49^ | Y | Y | Y | Y | N | U | Y | Y | Y | N | NA | N | N | Y |
| Butler-Laporte et al. (2022)^50^ | Y | Y | Y | Y | NA | Y | Y | Y | Y | N | Y | Y | Y | Y |
| Gill et al. (2020)^51^ | Y | Y | Y | Y | NA | N | Y | Y | Y | N | NA | N | Y | N |
| Sood et al. (2023)^52^ | Y | Y | Y | Y | NA | N | Y | N | Y | N | Y | N | Y | Y |
| Li et al. (2021)^53^ | Y | Y | Y | Y | NA | Y | Y | N | Y | N | Y | Y | Y | Y |
| Wang et al. (2021)^54^ | Y | Y | Y | Y | NA | Y | Y | N | Y | N | Y | Y | N | Y |
| Zhu et al. (2021)^55^ | Y | Y | Y | Y | Y | Y | Y | Y | Y | N | Y | Y | N | Y |

**Y=Yes, N=no, U=Unclear, NA=not applicable**

1. T**able S2.** MR strobe checklist for items related to reporting of the results and discussion according to MR-STROBE guidelines.

| items | 15 | 16 | 17 | 18 | 19 | 20 | 21 | 22 | 23 | 24 | 25 | 26 | 27 | 28 | TOTAL |
| --- | --- | --- | --- | --- | --- | --- | --- | --- | --- | --- | --- | --- | --- | --- | --- |
| Study | N of individuals | Summary statistics | Heterogeneity | Two-sample associations | Overlap | MR estimates | Plots | Validity* pleiotropy | Other | Sensitivity | Direction | Non-MR | Other plots | Limita-tions |  |
| Au Yeung et al. (2022)^31^ | Y | Y | Y | Y | N | Y | N | Y | Y | Y | Y | N | N | Y | 19 |
| Butler-Laporte et al. (2021)^32^ | Y | Y | Y | Y | N | Y | Y | Y | N | Y | N | N | N | Y | 21 |
| Li et al. (2021)^33^ | N | Y | N | N | N | Y | Y | Y | Y | Y | Y | Y | N | Y | 20 |
| Cui et al. (2021)^34^ | Y | Y | Y | Y | N | Y | Y | Y | Y | Y | Y | N | N | Y | 23 |
| Amin et al. (2021)^35^ | N | Y | Y | Y | N | Y | Y | Y | Y | Y | N | N | N | Y | 18 |
| Liu D et al. (2021)^36^ | Y | N | N | Y | N | Y | N | Y | Y | Y | N | N | N | Y | 16 |
| Patchen et al. (2021)^37^ | N | Y | N | Y | N | Y | Y | Y | Y | Y | N | N | N | Y | 19 |
| Qiu et al. (2023)^38^ | Y | U | N | Y | Y | Y | Y | Y | Y | Y | N | N | Y | Y | 22 |
| Hui et al. (2021)^39^ | Y | N | N | Y | N | Y | N | Y | N | N | N | N | N | Y | 14 |
| Mohus et al. (2022)^40^ | Y | Y | N | Y | N | Y | Y | Y | N | Y | Y | N | N | Y | 22 |
| Sobczyk et al. (2022)^41^ | N | Y | Y | Y | N | Y | Y | Y | Y | Y | Y | N | Y | Y | 21 |
| Daniel et al. (2022)^42^ | Y | Y | N | Y | N | Y | Y | Y | Y | Y | N | N | Y | Y | 23 |
| Zhou et al. (2022)^14^ | Y | Y | Y | Y | N | Y | Y | Y | Y | Y | Y | N | Y | Y | 24 |
| Palaniswamy et al.(2020)^43^ | Y | Y | Y | Y | N | Y | Y | Y | N | Y | N | Y | Y | Y | 22 |
| Liefaard et al. (2015)^44^ | N | Y | N | N | Y | Y | Y | N | N | Y | Y | N | N | Y | 18 |
| Kousathanas et al.(2022)^45^ | N | Y | Y | Y | N | Y | Y | Y | Y | Y | Y | N | Y | Y | 21 |
| Richardson et al.(2021)^46^ | N | Y | N | Y | N | Y | Y | Y | N | Y | Y | N | Y | Y | 19 |
| Gaziano et al. (2021)^47^ | Y | N | Y | Y | N | Y | Y | Y | Y | N | N | N | Y | Y | 16 |
| Zheng et al.(2022)^48^ | N | N | N | Y | N | Y | Y | N | N | N | N | N | Y | Y | 13 |
| Yang et al. (2022)^49^ | N | N | Y | Y | N | Y | Y | Y | Y | N | N | N | Y | Y | 16 |
| Butler-Laporte et al. (2022)^50^ | Y | Y | Y | Y | N | Y | N | Y | Y | Y | Y | N | Y | Y | 23 |
| Gill et al. (2020)^51^ | Y | N | Y | Y | N | Y | Y | Y | Y | Y | Y | N | N | Y | 18 |
| Sood et al. (2023)^52^ | Y | Y | Y | Y | N | Y | N | Y | Y | Y | N | N | Y | Y | 19 |
| Li et al. (2021)^53^ | Y | Y | Y | Y | N | Y | Y | Y | Y | Y | Y | N | N | Y | 22 |
| Wang et al. (2021)^54^ | Y | Y | Y | Y | Y | Y | Y | Y | Y | Y | Y | N | Y | Y | 23 |
| Zhu et al. (2021)^55^ | Y | N | Y | Y | N | Y | Y | Y | Y | N | Y | N | Y | Y | 22 |

**Y=Yes, N=no, U=Unclear, NA=not applicable.**

1. **Table S3.** Randomized controlled studies on the impact of vitamin D on COVID-19 disease.

| **Author, Year, Country** | **Study Design/study population** | **Characteristics** | **Intervention** | **Clinical Variables Outcomes** | **Results** | **Limitations** |
| --- | --- | --- | --- | --- | --- | --- |
| ***Vitamin D*** | | | | | | |
| Alcala-Díaz et al.(2021)^69^  Spain | Quasiexperimental clinical trial  Follow-up period: 30 days  N=537  Patients with clinical symptoms of acute respiratory infection by COVID-19, radiographic signs of viral pneumonia, and positive PCR | Intervention Group (N=79), aged 69 ± 15 years, 53% males  Control Group (N=458), aged 67 ± 16 years, 60% males | Supplementation with oral calcifediol (0.266 mg/capsule, 2 capsules upon admission, and then one capsule on days 3, 7, 14, 21, and 28) in the intervention group  No supplementation with calcifediol in the control group.  Both groups received treatment for Covid-19 | Primary:  1. Mortality within the first 30 days after admission  Secondary:  1. Complete blood count  2. Coagulation study (d-dimer)  3. Kidney and liver function  4. LDH  5. Ferritin  6. PCR  7. IL6  8. Oxygen saturation  9. Chest X-ray  10. ARDS (Acute Respiratory Distress Syndrome)  11. CURB-65 (pneumonia severity) | The intervention group had a lower risk of death during hospitalization, while the control group had lower SpO2 at admission, higher PCR levels, higher blood urea nitrogen, and higher rates of CURB65 ≥ 3 and moderate to severe ARDS at admission. For the other variables, no significant differences were found | This was a quasiexperimental study conducted on patients admitted during the first wave, where treatment administration was not randomized and was based on the clinical judgment of healthcare professionals, which may introduce potential unidentifiable residual confounding. Data such as the onset date of symptoms before admission, patients' body mass index, the presence of other comorbidities, and the rate of acute kidney injury development, among others, were missing in most patients and could not be included (as this data was derived from preexisting medical records). Finally, postdischarge follow-up events were not considered due to a lack of such information |
| Murai et al. (2021)^70^  Brazil | Multicenter, double-blind, parallel-group, randomized (1:1), and placebo-controlled clinical trial  Follow-up period: June-October 2020  N=240  Patients with moderate to severe Covid-19 (diagnosed by PCR or antibody test) (flu-like symptoms, respiratory rate >24/min, oxygen saturation <93%, or risk factors for complications such as heart disease, diabetes...) | Intervention Group (N=117), aged 56.5 (± 13.8) years, 58.8% males, and 41.2% females  Control Group (N=118), aged 56 (± 15) years, 53.4% males, and 46.6% females | Supplementation with a single oral dose of 200,000 IU of VD3 dissolved in peanut oil solution in the intervention group  Supplementation with 10 ml of peanut oil solution in the control group | Primary:  1. Duration of hospital stay  Secondary:  1. In-hospital mortality  2. Number of patients admitted to the ICU  3. Number of patients requiring mechanical ventilation  4. Duration of mechanical ventilation  5. Serum levels of 25-hydroxyvitamin D, total calcium, creatinine, C-reactive protein, D-dimer | There were no significant differences between the VD3 and placebo groups for: duration of hospital stay, mortality, admission to the ICU, need for mechanical ventilation, total calcium, creatinine, CRP, or D-dimer. The mean (SD) of 25-hydroxyvitamin D significantly increased after a single high dose of VD3 compared to placebo | Limitations of this study include the small sample size, which may exclude significant differences between the two groups. Caution should be exercised when generalizing results as the patient sample is small, and there was variability in initial vitamin D levels. The results may be affected by the heterogeneity of the sample and treatments (patients had different comorbidities and, therefore, different treatments). The administration of vitamin D is late, and it would be necessary to study its early or preventive administration in this disease. The deficiency of 25-hydroxyvitamin D3 was lower than in other cohorts |
| Sabico (2021)^71^  Saudi Arabia | Randomized 1:1 multicenter retrospective clinical trial  Follow-up period: 2 months  N=69  Adult male and female patients aged 20 to 75 years with a positive diagnosis of SARS-CoV-2 confirmed by PCR (not more than 3 days before inclusion) and experiencing mild to moderate symptoms | Intervention Group (5000 IU) (N=36), aged 46.3 (± 15.2) years, 58% males, and 42% females  Control Group (1000 IU) (N=33), aged 53.5 (± 12.3) years, 40% males, and 60% females | Supplementation with Ultra-D® 5000 IU containing 125 µg of cholecalciferol (vitamin D3) in the intervention group. Supplementation with Vita-D® 1000 IU containing 25 µg of cholecalciferol in the control group  Both supplements were taken orally daily for 2 weeks  They were asked to return after one week (Day 7) for symptom monitoring | Primary:  1. Time to resolution of symptoms  Secondary:  1. Changes in metabolic profile  2. Days to discharge  3. Admission to the ICU  4. Mortality | The number of days to resolve cough was significantly shorter in the 5000 IU group than in the 1000 IU group, and the same trend was observed for the loss of taste. No adverse events related to treatment were reported in either of the groups. There was a significant decrease in BMI in both groups. In the 1000 IU group, there was a significant increase in hematocrit, lymphocytes, prothrombin, and ferritin  On the other hand, in the 5000 IU group, there was a significant increase in neutrophils, urea levels, and 25(OH)D levels | Limitations of the study include the risk of bias due to the lack of blinding and the small sample size |
| Annweiler et al. (2021)^72^  France | Quasiexperimental retrospective study  Follow-up period: 3 months or death  N=95  Geriatric patients with COVID-19 diagnosed by RT‒PCR and/or chest computed tomography; with usual serum measures available; and availability of vital status 3 months after COVID-19 diagnosis | Intervention Group (N=67), aged 87.7 ± 5.4 years, 56.7% females and 43.3% males  Control Group (N=28), aged 88.6 ± 5.7 years, 28.6% females and 71.4% males  Baseline characteristics similar between groups. | Supplementation included either 50,000 IU of vitamin D3 per month, or 80,000 IU, or 100,000 IU, or 200,000 IU of vitamin D3 every 2 or 3 months. One patient was supplemented with 800 IU of vitamin D3 per day. None received D2 or intramuscular supplements. All COVID-19 participants had received oral vitamin D3 supplements before hospitalization, and/or during hospitalization, and/or upon hospital discharge  No supplementation in the control group before or after COVID-19 diagnosis | Primary:  1. All-cause mortality at 3 months | Mortality at 3 months was lower in the intervention group compared to the control group | Limitations of the study include the small study population, potential residual confounding factors, and the less robust quasiexperimental design compared to a randomized controlled trial |
| Elamir et al. (2022)^73^  USA | Randomized, open-label 1:1 clinical trial  N=50  Consecutive adult patients hospitalized with COVID-19 | Intervention Group (N=25), aged 69 ± 18 years, 48% males and 52% females  Control Group (N=25), aged 64 ± 16 years, 52% males and 48% females | Supplementation with 0.5 μg of calcitriol daily for 14 days or until hospital discharge in the intervention group  No supplementation in the control group  For both groups, the rest of the care was determined by the primary team and may include treatment with remdesivir (200 mg for one day followed by 100 mg for 4 days), dexamethasone (6 mg daily for 10 days), or convalescent plasma, as well as supplementary oxygen | Primary:  1. Evaluate respiratory status and oxygen requirement  2. Ratio of peripheral arterial oxygen saturation to fraction of inspired oxygen (SpO2/FIO2)  Secondary:  1. Duration of hospital stay  2. ICU admission  3. Mortality  4. Rehospitalization | The control group had a higher mean increase in the SpO2/FIO2 ratio compared to the intervention group. The mean duration of hospital stay, ICU admission, oxygen requirements, rehospitalizations, and mortality were higher in the intervention group compared to the control group | Limitations of the study include the lack of a placebo control group and blinding, absence of vitamin D level measurements, a higher number of elderly and comorbid patients in the control group, and a limited study population |

1. **Table S4.** Randomized controlled studies on the impact of micronutrients other than vitamin D on COVID-19 disease.

| **Author, Year, Country** | **Study Design/study population** | **Characteristics** | **Intervention** | **Clinical Variables Outcomes** | **Results** | **Limitations** |
| --- | --- | --- | --- | --- | --- | --- |
| ***Vitamin C*** | | | | | | |
| Thomas et al. (2021)^78^  USA | Randomized Clinical Trial  Study  Follow-up period: 28 days  N=214  Ambulatory Patients with COVID-19 (confirmed diagnosis via PCR) | Intervention Group (N=164)  1. Ascorbic Acid Only: N=48, 45.6 (±15) years, 66.8% females and 33.2% males  2. Zinc Only: N=58, 44.1 (±14.8) years, 63.8% females and 36.2% males  3. Both: N=58, 48.7 (±14.3) years, 53.4% females and 46.6% males  Control Group: N=50, 42 (±14.6) years, 62% females and 38% males | The patients were randomized into 4 groups to receive ascorbic acid for 10 days:  1. Ascorbic acid 8 mg/day (divided into 3 doses)  2. Zinc gluconate (50 mg/day) at night  3. Both treatments together  4. No treatment (control group) | Primary:  1. Days to 50% reduction in 4 symptoms (fever, cough, difficulty breathing, fatigue), measured using a score (0-4 points)  2. Days to complete symptom remission (score=0)  Severity of symptoms on the fifth day (score: 0-4)  Secondary:  1. Death  2. Hospitalization  3. Adverse Effects  4. Medication | There were no significant differences among the four groups | There was no placebo control group; instead, there was a standard treatment control group. The patients were not blinded. They were recruited from a single center, limiting the extrapolation of results to other populations  Groups with higher susceptibility, such as older individuals, were underrepresented in the study, and the results cannot be widely generalized |
| Xing et al. (2021)^79^  China | Quasiexperimental clinical trial  N=91  Patients with COVID-19 | Intervention Group (N=31), 39 years, 68% males and 32% females  Control Group (N=60), 35.63 years, 75% males and 25% females | Supplementation with a dose of 100 mg/kg/day intravenous vitamin C (VC) in the intervention group  No supplementation in the control group  Plasma concentrations of VC were measured before dosing and 5 to 15 days after dosing  One to three fasting blood samples (2 ml in tubes containing ethylene-diaminetetraacetic acid) were collected from each volunteer | Primary:  1. Creatinine levels, alanine aminotransferase, and aspartate aminotransferase  Secondary:  1. Stability of VC in blood  2. Concentration of VC in plasma | Patients with COVID-19 had a significant deficiency of VC. Following VC administration, the average plasma concentration increased to levels similar to the healthy volunteer group | Only healthy volunteers were enrolled to eliminate the effect of smoking on VC concentrations, and it was a quasiexperimental study involving only 31 COVID-19 patients, rather than a multicenter randomized controlled trial |
| Jamali-Moghadam-Siahkali et al. (2021)^80^  Iran | Randomized Clinical Trial  N=60  Patients with severe COVID-19 | Intervention Group (N=30), 57.53 (±18.27) years, 50% male and 50% female  Control Group (N=30), 61 (±15.90) years, 50% male and 50% female  Baseline characteristics were similar between the groups | Supplementation of 1.5 g intravenous vitamin C (VC) every 6 hours for 5 days in the intervention group  No supplementation in the control group. Both groups were treated with oral lopinavir/ritonavir 400/100 mg twice a day and a single dose of oral hydroxychloroquine on the first day of hospitalization | Primary:  1. Mortality  2. Duration of hospitalization  3. Admission to ICU  Secondary:  1. Improvements in SpO2 and vital signs  2. Severity score of symptoms | The intervention group had a lower frequency of fever and myalgia symptoms, their average hospital stay was significantly shorter, and the SpO2 on the 3rd day of admission was higher  However, there were no significant differences between the two groups in terms of ICU stay, intubation, and mortality | Control group, but apparently without a placebo  Differences between the groups at the outset in terms of the presentation of some symptoms  Limited study population size  Nonblinded study |
| Kumari et al. (2020)^81^  Pakistan | Randomized controlled trial with a control group  Follow-up period: 28 days  N=150 Patients in the severe COVID-19 unit | Intervention Group (N=75)  Control Group (N=75)  52 (± 11) years old  (56.9% males and 43.1% females)  Baseline characteristics were similar between the groups | Supplementation with intravenous vitamin C (VC) at a dose of 50 mg/kg/day in the intervention group, along with standard therapy  No supplementation in the control group  Both groups were treated with antipyretics, dexamethasone, and prophylactic antibiotics | Primary:  1. Respiratory rate  2. Oxygen saturation  3. PCR  4. LDH  Secondary:  1. Resolution of symptoms  2. Treatment  3. Hospital stay  4. Mechanical ventilation  5. Mortality | Patients in the intervention group, compared to those in the control group, experienced symptom resolution earlier and had a shorter average hospital stay. There were no statistically significant differences between the groups in relation to the other outcome variables studied | Since it was a single-center study, the generalizability of the results may be limited  No investigator blinding  No placebo control group |
| Majidi et al. (2021)^82^  Iran | Double-blind randomized clinical trial  Follow-up period: 14 days  N=100  Critically ill patients aged 35 - 75 years hospitalized in the ICU with COVID-19 infection and an indication for parenteral nutrition | Intervention Group (N=31)  Control Group (N=69)  Aged 59.42 (± 15.07) years old | Supplementation with one 500 mg vitamin C (VC) capsule per day (added to their enteral nutrition) for 14 days in the intervention group  The control group received the same enteral nutrition without vitamin C supplementation  Supplementation was initiated within 24 hours of ICU hospitalization through enteral nutrition | Primary:  1. Survival  Secondary:  1. Blood biomarkers  2. Serum electrolyte levels (serum levels of Na, Ca, and P)  3. Arterial blood gas parameters  4. Hospital stay | Higher survival rate in the intervention group | We did not measure serum levels of vitamin C, so the patients' vitamin C status was not adjusted for before the study; due to the critical condition of the patients, the VC dose was low |
| ***Zinc*** | | | | | | |
| Thomas et al. (2021)^78^  USA | Randomized clinical trial  Follow-up period: 28 days  N=214  Outpatients with confirmed COVID-19 diagnosis by PCR test | Intervention Group (N=164)  1.Ascorbic acid alone N=48, aged 45.6 (±15) years, 66.8% females and 33.2% males  2.Zinc alone N=58, aged 44.1 (±14.8) years, 63.8% females and 36.2% males.  3.Both, N=58, aged 48.7 (±14.3) years, 53.4% females and 46.6% males  Control Group (N=50), aged 42 (± 14.6) years, 62% females and 38% males | Patients were randomized into 4 groups to receive ascorbic acid for 10 days:  1.Ascorbic acid 8 mg/day (divided into 3 doses)  2.Zinc gluconate (50 mg/day, at night)  3.Both treatments together  4.No treatment (control group) | Primary:  1. Days to 50% reduction in 4 symptoms (fever, cough, shortness of breath, fatigue), measured using a score (0-4 points)  2. Days to complete symptom remission (score=0)  3. Severity of symptoms on the fifth day (score: 0-4)  Secondary:  1. Death  2. Hospitalization  3. Adverse effects  4. Medication | There were no significant differences among the four groups | There was no placebo control group, only a standard treatment group. The patients were not blinded. Recruitment was done from a single center, limiting the generalizability of the results to other populations. Groups with higher susceptibility, such as older individuals, were underrepresented in the study, and the results may not be widely applicable |
| Patel et al. (2021)^83^  Australia | Double-blind, randomized controlled trial with control group  Follow-up period: 7 days  N=33  Hospitalized COVID-19 patients with SpO2 of 94% or less | Intervention Group (N=15), aged 59.8 ± 16.8 years, 73.3% males and 26.7% females  Control Group (N=18), aged 63.8 ± 16.9 years, 55.5% males and 44.5% females | Supplementation involved zinc chloride (ZnCl2) diluted in 250 ml of normal saline solution and infused through a peripheral intravenous access for 3 hours at a dose of 0.5 mg/kg/day (elemental zinc concentration, 0.24 mg/kg/day) for a maximum of 7 days in the intervention group  Placebo supplementation with saline solution for the control group | Primary:  1. Oxygenation level expressed as oxygen flow in nonventilated patients  2. PaO2/FiO2 ratio in ventilated patients  Secondary:  1. Adverse effects | The primary outcomes could not be assessed. No adverse effects were observed with the intervention, but irritation at the infusion site was noted. HDIVZn increased serum zinc levels above the deficiency threshold, while with placebo, they remained below this value | The study had a limited sample size, and there were not enough blood samples collected to extract the desired results |
| Abd-Elsalam et al. (2020)^84^  Egypt | Randomized clinical trial  Follow-up period: 28 days  N=191  Patients with confirmed diagnosis of Covid-19 | Intervention Group (N=96), aged 43.48 ± 14.62 years, 54.2% males and 45.8% females  Control Group (N=95), aged 43.64 ± 13.17 years, 67.4% males and 32.6% females  Baseline characteristics were similar between the groups | Supplementation included a dose of 220 mg of zinc sulfate (50 mg of elemental zinc) twice a day in the intervention group  No zinc supplementation in the control group  Both groups received standard care for COVID-19 infection for 15 days, including hydroxychloroquine at a dose of 400 mg twice a day on the first day, followed by 200 mg twice a day for 5 days | Primary:  1. 28-day recovery  2. Mechanical ventilation  3. Mortality  Secondary:  1. Hospital stay | There were no significant differences in supplementation | The control group, however, appeared to lack a placebo. Serum zinc levels were not measured before, during, or after treatment in this clinical trial. Zinc absorption may be limited by a diet high in phytate and other medications |
