# Supplementary material for: Micronutrients, Vitamin D, and Inflammatory Biomarkers in COVID-19: A Systematic Review and Meta-analysis of Causal Inference Studies
Source: Nutr Rev. 2024 Oct 24;83(7):e1383–405. doi: 10.1093/nutrit/nuae152 (PMC12166185; doi:10.1093/nutrit/nuae152)
Supplement: nuae152_Supplementary_Data [file nuae152_supplementary_data.zip › nuae152_Supplementary_Data/Alcalá-Santiago et al. Figures Nut Rev 4.docx]

**Figure 1. Prisma 2020 flow diagram of the study search and selection process**

**
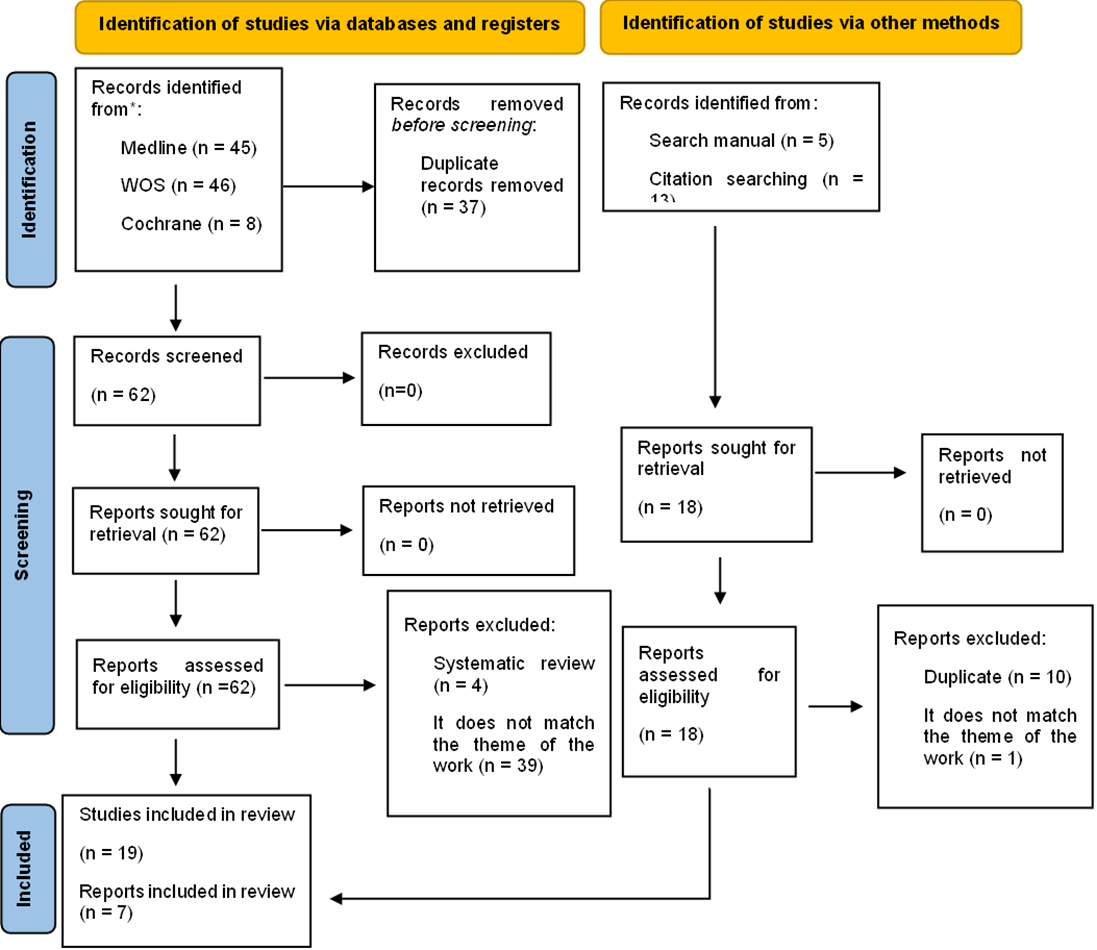
**

**Figure 2. Exposure-outcome associations.**


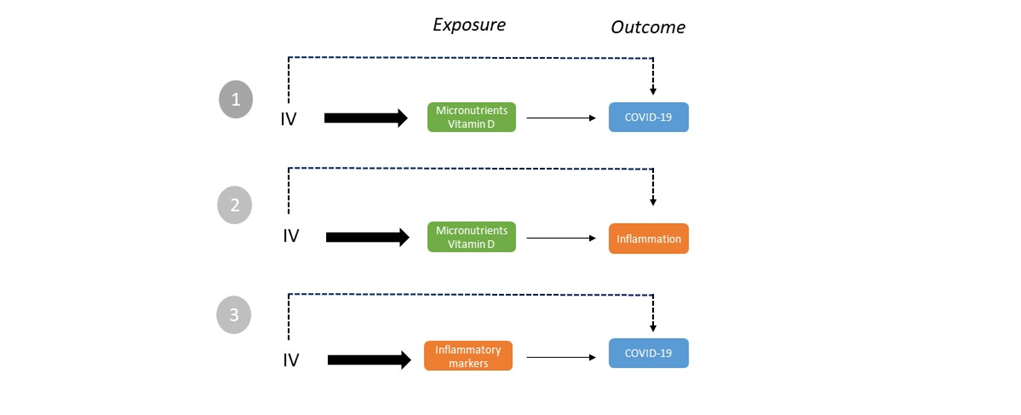


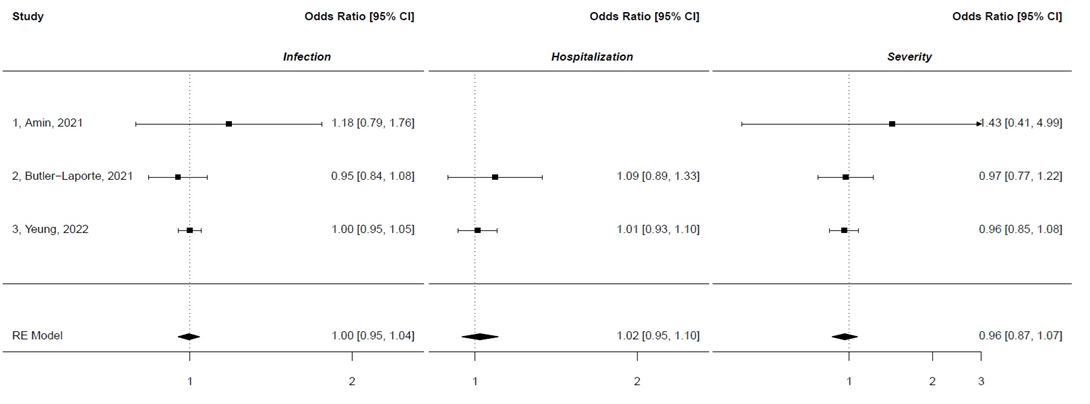
**Figure 3. Meta-analyses of studies reporting results on VD and COVID-19 disease outcomes**

**A B C**


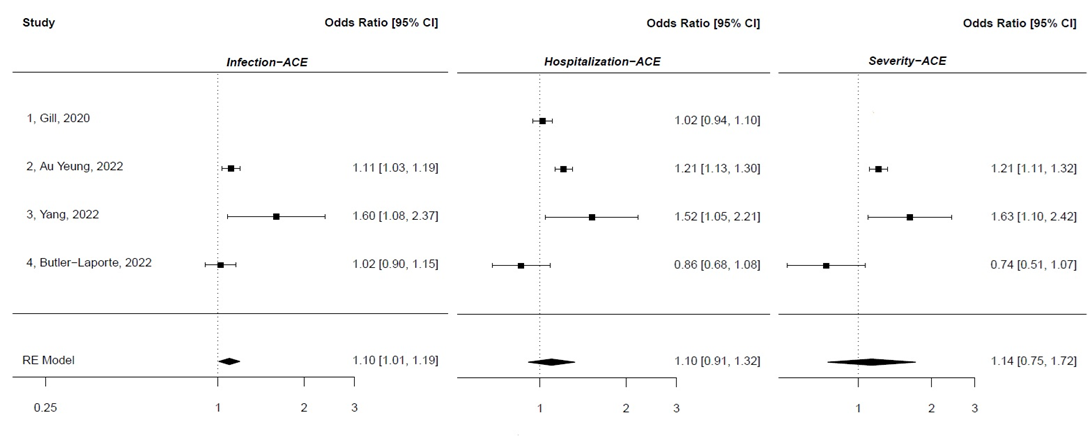
**Figure 4. Meta-analyses of studies reporting results on ACE2 and COVID-19 disease outcomes.**

**A B C**
